# Domains and processes for institutionalizing evidence-informed health policy-making: a critical interpretive synthesis

**DOI:** 10.1186/s12961-022-00820-7

**Published:** 2022-03-04

**Authors:** Tanja Kuchenmüller, Laura Boeira, Sandy Oliver, Kaelan Moat, Fadi El-Jardali, Jorge Barreto, John Lavis

**Affiliations:** 1 Research for Health, Science Division, World Health Organization, Geneva, Switzerland; 2 Instituto Veredas, São Paulo, Brazil; 3 Social Research Institute, University College London, London, United Kingdom; 4 Faculty of Humanities, University of Johannesburg, Johannesburg, South Africa; 5 McMaster Health Forum/WHO Collaborating Centre for Evidence-Informed Policy, McMaster University, Hamilton, ON Canada; 6 Department of Health Evidence and Impact, McMaster University, Hamilton, ON Canada; 7 Knowledge to Policy (K2P) Center/WHO Collaborating Centre for Evidence-Informed Policy and Practice, American University of Beirut, Beirut, Lebanon; 8 Department of Health Management and Policy, American University of Beirut, Beirut, Lebanon; 9 Oswaldo Cruz Foundation (Fiocruz), Brasília, Brazil

**Keywords:** Evidence-informed policy, Institutionalization, Institutional capacity, Knowledge translation, Knowledge translation platform, Sustainability, Conceptual framework, Definition

## Abstract

**Background:**

While calls for institutionalization of evidence-informed policy-making (EIP) have become stronger in recent years, there is a paucity of methods that governments and organizational knowledge brokers can use to sustain and integrate EIP as part of mainstream health policy-making. The objective of this paper was to conduct a knowledge synthesis of the published and grey literatures to develop a theoretical framework with the key features of EIP institutionalization.

**Methods:**

We applied a critical interpretive synthesis (CIS) that allowed for a systematic, yet iterative and dynamic analysis of heterogeneous bodies of literature to develop an explanatory framework for EIP institutionalization. We used a “compass” question to create a detailed search strategy and conducted electronic searches to identify papers based on their potential relevance to EIP institutionalization. Papers were screened and extracted independently and in duplicate. A constant comparative method was applied to develop a framework on EIP institutionalization. The CIS was triangulated with the findings of stakeholder dialogues that involved civil servants, policy-makers and researchers.

**Results:**

We identified 3001 references, of which 88 papers met our eligibility criteria. This CIS resulted in a definition of EIP institutionalization as the “process and outcome of (re-)creating, maintaining and reinforcing norms, regulations, and standard practices that, based on collective meaning and values, actions as well as endowment of resources, allow evidence to become—over time—a legitimate and taken-for-granted part of health policy-making”. The resulting theoretical framework comprised six key domains of EIP institutionalization that capture both structure and agency: (1) governance; (2) standards and routinized processes; (3) partnership, collective action and support; (4) leadership and commitment; (5) resources; and (6) culture. Furthermore, EIP institutionalization is being achieved through five overlapping stages: (i) precipitating events; (ii) de-institutionalization; (iii) semi-institutionalization (comprising theorization and diffusion); (iv) (re)-institutionalization; and (v) renewed de-institutionalization processes.

**Conclusions:**

This CIS advances the theoretical and conceptual discussions on EIP institutionalization, and provides new insights into an evidence-informed framework for initiating, strengthening and/or assessing efforts to institutionalize EIP.

**Supplementary Information:**

The online version contains supplementary material available at 10.1186/s12961-022-00820-7.

## Background

### Evidence-informed policy-making (EIP): challenges to the practical application

Throughout the last three decades, EIP—defined as the systematic and transparent use of the best available data, research and other forms of evidence (such as modelling, evaluations or behavioural/implementation research) depending on the nature of the decision-making process [1–4]—has increasingly been recognized as an important concept supporting governments in improving the effectiveness, efficiency and perceived legitimacy of decision-making [5, 6]. It has overall become a normative notion, the “zeitgeist“, of how policy formulation and implementation ought to be undertaken to make best use of resources and increase civic trust [7, 8]. Despite wide agreement that evidence-informed policy is both desirable and feasible, the rhetoric seems to be stronger than the practical application [5].

Indeed, research often goes unused. Some studies are a poor fit with society’s important problems, some offer partial reporting, and others remain unpublished [9]. In addition, weak engagement between researchers and policy-makers, poor communication of relevant research, absence of supportive organizational systems, and a lack of capacity among decision-makers to access, appraise and apply research are frequently referenced barriers [10]. Also, knowledge translation (KT)^1^ processes are, at times, still designed based on the assumption that the use of evidence in health policy-making can be reduced to a linear, problem-solving and technical-rational process in which “objective” scientific research is easily applied in an instrumental manner [5, 11]. Fostering the use of evidence in policy is, however, a complex, multifaceted [12] and inherently political process [13] where a diversity of factors influence the way evidence can shape policy [14], requiring interactive and systemic approaches [15]. Many potential meanings and typologies of evidence use in policy exist [3, 16–18], which are often summarized in three main approaches [19]: instrumental (direct use to influence decisions), conceptual (indirect use to change understanding) or symbolic (political or persuasive use to legitimize predetermined positions). This paper takes a programmatic health planning approach according to which evidence use serves the achievement of goals pursued by an administrative body (such as the ministry of health, or its departments) aligned with societal aspirations [3].

### WHO’s global efforts in promoting and institutionalizing EIP

Recognizing the need to strengthen the research–policy nexus, in 2005, countries requested that WHO “establish mechanisms to transfer knowledge in support of evidence-based public health and health-care delivery systems and evidence-based health-related policies” [20, p. 3]. As a response, WHO launched the Evidence-informed Policy Network (EVIPNet), which is a global network and community of people that share a vision to see, and collaboratively engage in efforts to support, a world in which high-quality, context-sensitive evidence routinely informs health decision-making processes to improve health outcomes [21, 22].

While the majority of KT capacity-building initiatives have centred on individual behaviour and cognitive changes, there is an increasing interest in organizational capacity and larger systems changes [23–25]. For instance, beyond the provision of trainings, technical assistance and mentoring, EVIPNet supports its member countries in establishing so-called knowledge translation platforms (KTPs), which are partnerships comprising national researchers, policy-makers and the civil society, serving as organizational knowledge brokers. These KTPs display different design features and organizational models depending on the organization’s function and context [26]. Organizational knowledge brokers, such as KTPs, with the mandate to interconnect evidence to the policy processes, have been suggested as a promising institutional mechanism to bridge the gap between research and policy communities that are often deeply separated in terms of culture, process, language, time frames and incentives [27, 28].

The dual EVIPNet approach of capacity-building and EIP institutionalization was strategically chosen, based on the knowledge that initiatives which cannot be sustained lead to a substantial waste in human and financial investments, as well as a decline in stakeholder interest in engaging in similar projects in the future [29]. Institutionalization in this context can be understood as a “process by which a set of activities becomes an integral and sustainable part of a formal system” [30, p. 2], leading to stability and durability [31] or staying power [12].

Despite the broad acknowledgements for the need and the growing establishment of organizational knowledge brokers for health policy-making, our practical and theoretical understanding about their creation, operationalization and in particular how they can, once in place, be sustained remains overall limited [12–14, 23, 32]. A few recent papers have addressed organizational aspects of units in support of EIP [12, 30, 31, 33–35], such as Zida et al., who conducted a case study on the institutionalization of a rapid response unit in Burkina Faso [30, 35], Al Sabahi et al. with their critical interpretative synthesis on approaches to establishing policy support organizations [34], and Koon et al.’s scoping review on institutionalizing knowledge for health policy in low- and middle-income countries [31]. The time is now ripe to look across and beyond case studies to consider EIP institutionalization more comprehensively by analysing both theoretical papers and empirical studies. The development of an evidence-informed theoretical framework is therefore suggested to provide a deeper understanding and integrate the key features to be considered for a systematic approach towards creating and maintaining EIP institutionalization. This framework intends to inform and add value to the work of both KT researchers and practitioners, in particular EVIPNet member countries.

Based on a systematic knowledge synthesis of the published and grey literature, this article tries to offer, as a first step, a preliminary definition of the term institutionalization, which will be further refined throughout this paper. As a second step, this paper aims to provide an evidence-informed theoretical framework identifying the domains that are likely key for EIP institutionalization, as well as, as a third step, the processes of EIP institutionalization.

## Methodology

A critical interpretive synthesis (CIS) approach was conducted, supplemented by a stakeholder engagement process, which enabled us to triangulate and refine our findings based on the views of countries embarking on the establishment and operationalization of organizational knowledge brokers (for a similar combination of CIS with an integrated KT approach see [6]).

Following the CIS methodology, this study applies a two-pronged approach: a systematic literature review with explicit, structured methods to search the indexed literature electronically, which was supplemented with purposive sampling and inductive analysis, to ensure that the final sample of included papers was theoretically rich and relevant to the development of theoretical constructs based on the emerging themes and concepts. The aim was to find new concepts rather than papers that reiterated ideas already identified in previously screened literature. Furthermore, additional purposive sampling of included papers, and additional purposively identified papers to fill conceptual gaps (as needed), was undertaken until theoretical saturation was reached [36, 37].

### Review questions

According to the CIS methodological standards, a “compass”^2^ question and related sub-questions were developed, which were used to search and identify relevant literature for the development of the EIP institutionalization framework [37–39]. The following compass questions and sub-questions were identified, subject to constant modification in an iterative manner, for example moving from the institutionalization of “policy supports for the use of research evidence“ (such as KTPs) to the “EIP“ institutionalization, realizing that the former is but one element of the overall EIP institutionalization process (see “The six domains of EIP institutionalization” section).

Compass question: What are the features of and approaches to institutionalizing EIP?

Sub-questions:

1. What definitions of and approaches to institutionalizing the use of research evidence by health policy-makers exist?
2. What are the domains of institutionalizing health policy-makers’ use of research evidence?
3. What is the process of institutionalizing health policy-makers’ use of research evidence?

### Literature search

Following the CIS methodology, a multistep approach was undertaken to create a sample of studies to be included in the synthesis.

#### Electronic searches

Firstly, an explicit and structured approach was applied, following the principles of a conventional systematic review. A broad search of the literature was conducted using a combination of keywords. Based on the compass question, a table of Boolean-linked keywords and synonyms was developed and a range of search strategies tested. The search strategy was developed in consultation with a library scientist at Biblioteca Regional de Medicina (BIREME-Regional Library of Medicine). The selected search string comprises three key terms: “institutionalization” AND “knowledge translation” AND “policy-making”. Each of the terms was complemented by related search terms (e.g. “knowledge translation” by “knowledge exchange”, “knowledge use”, etc.) and takes account of different spellings (e.g. “policy-makers”, “policymakers”, policy makers”). Sensitivity rather than specificity was aimed for [40]. The search strategy included various electronic databases and sources: PubMed, Social Systems Evidence, Health Systems Evidence, Virtual Health Library, Web of Science, Embase, CINAHL, Cochrane Library, Google and Google Scholar (see Additional file 1 for the search strategies adapted to the search interface and functionality of each of the databases).

Searches were undertaken between 31 December 2020 and 21 January 2021. An unlimited search was conducted for geographical location (high-, middle-, low-income countries) and languages.

**Fig. 1:**
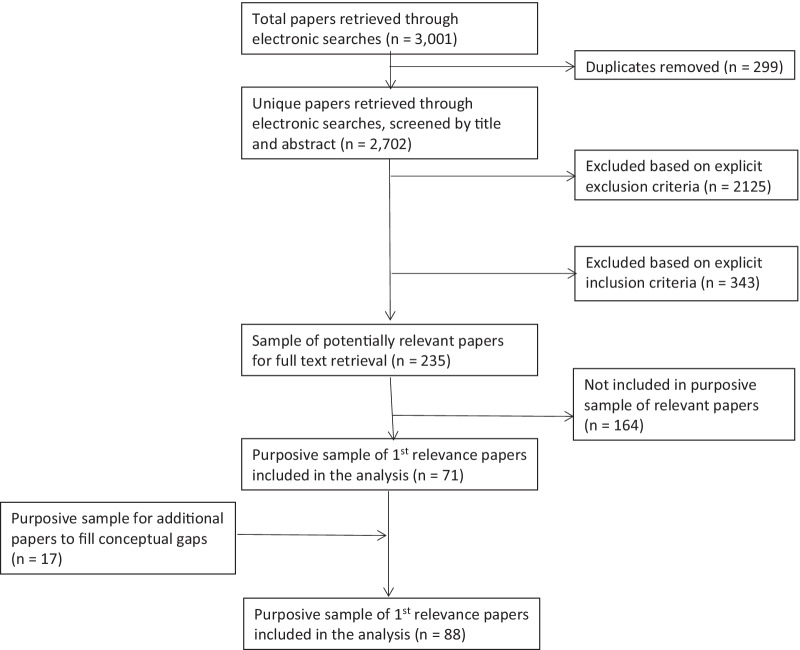
PRISMA flowchart

In addition, literature was purposively identified based on previous work on institutionalization conducted by the authors to fill conceptual gaps that emerged from the mapping of relevant articles through an inductive constant comparative approach. For the full overview of included and excluded papers, see the PRISMA (Preferred Reporting Items for Systematic Reviews and Meta-Analyses) flowchart (Fig. 1).

#### Developing and applying selection criteria

Based on a sample of papers identified through the electronic searches, inclusion and exclusion criteria were developed by two independent reviewers (TK and LB) and iteratively amended.

Titles and abstracts of all references identified by the electronic searches were reviewed. Each reference was assessed in duplicate. First, the exclusion criteria were applied. Secondly, on the remaining pool of papers, the inclusion criteria were used to identify the pool of “potentially relevant“ papers. Papers were excluded if, for instance, they focused on primary or secondary research production, or on KT at the level of individuals (e.g. promoting behaviour change). (For the detailed exclusion and inclusion criteria, see Additional file 2.)

For the pool of “potentially relevant“ papers, the full text was retrieved and reviewed to make a final assessment whether the papers initially included were pertinent to the CIS based on the compass question and the principal aims of the review. Papers considered relevant were included in the sample frame from which we retrieved our purposive sample for the synthesis.

#### Conceptual mapping/data extraction

The relevant papers were mapped using a standardized form (see Additional file 3). Data extraction was carried out by one reviewer and double-checked by a second reviewer (TK, LB).

#### Purposive sampling

The mapping exercise enabled detection of areas that were conceptually rich. Papers for which, based on the mapping exercise, saturation was achieved were not included for data extraction. Both the mapping exercise and the data extraction allowed us to identify areas where literature was lacking. This informed the selection of a purposive sample of relevant papers.

#### Quality appraisal

The CIS relies on the use of theoretical relevance rather than methodological characteristics as a criterion to identify the “quality“ of papers. In other words, the quality of the literature was judged as the extent to which it contributes to conceptual development. The threshold of inclusion was informed by expertise and judgement rather than being articulated a priori [37, 39, 41].

### Stakeholder engagement

The CIS was supplemented with a stakeholder engagement process (stakeholder dialogue) [6] to triangulate the findings and validate our understanding. At two multicountry meetings of the EVIPNet [42, 43], the national champions of 20 EVIPNet country teams^3^ (i.e. the entities leading the WHO-supported EVIPNet activities at the country level) were invited to work in groups to brainstorm on and identify what constitutes EIP institutionalization. These stakeholders were chosen based on their understanding of EIP and their ability to champion the introduction of new processes to ensure that concepts and themes developed through the CIS would resonate with those in positions to effect and initiate institutionalization processes. We convened 36 participants in total, namely 17 health experts/civil servants of background institutions and agencies, 17 policy-makers (from ministries of health) and two external (academic) researchers. The engagement approach was based on three steps: First, TK provided the rationale for network members going beyond organizational capacity-building and sustaining their efforts through EIP institutionalization. Second, the EVIPNet members were asked to consider in small groups the theoretical aspects as well as the local applicability and feasibility of institutionalizing EIP in their countries. As a third step, outputs from the seven small groups were reported and discussed in plenary as well as captured in the related meeting reports (for more information on the process and findings of the stakeholder engagement process as well as the full list of stakeholders who participated in this exercise, see [42, pp. 21–3, 43, pp. 32–4]).

### Data extraction, synthesizing and integrating findings

TK and LB extracted data from each of the included articles by using a data extraction sheet that was piloted as a first step. For the piloting, both reviewers filled in the pro forma sheet with the findings of a few papers only to test its suitability. No changes to the pro forma needed to be undertaken.

A constant comparative method was applied to develop an explanatory framework on EIP institutionalization. This included an iterative approach focusing on:

- Developing common themes and concepts based on the data extracted from each paper;
- Developing a theoretical construct based on the emerging themes and concepts;
- Conducting additional purposive sampling of included papers and/or conducting additional purposive searches to fill conceptual gaps (as required) until theoretical saturation was reached; and
- Consulting the reports of the stakeholder engagement groups to verify any additions or inconsistencies between the findings of the CIS and their contributions.

## Results

The database search yielded 3001 articles. When duplicates were removed, 2702 unique citations were assessed, and 235 articles were identified as potentially relevant. After full-text review of these, 164 citations were excluded. A purposive sample of 71 citations was selected based on two guiding principles: on the one hand, their relevance relating to any of the three review sub-questions and, on the other hand, theoretical saturation. An additional 17 papers filled conceptual gaps. In total, 88 papers were considered as highly relevant, of which 43 citations presented definitions of EIP institutionalization, 43 citations presented relevant frameworks (7 process frameworks, 31 domain frameworks and 5 both process and domain frameworks), 24 presented theories and 47 presented other relevant data on domains and process.

- The majority of articles included (51%; *n* = 45) were published between 2015 and 2020.
- Many articles focused on EIP and KT (36%; *n* = 32), followed by national immunization technical advisory groups/health technology assessment/health impact assessment experiences and so on (14%; *n* = 12).
- Many articles (49%; *n* = 43) had a specific geographical focus (country or region), with some focusing on the global level or multiple regions (21%; *n* = 18).

Stakeholders’ perspectives were used to triangulate and validate the literature review findings, with similar content being found both in the CIS and in the reports of the stakeholder dialogues.

While initially, when designing the CIS, it had not been foreseen to exclusively focus on health (neither the initial compass question nor the search strategy was therefore limited to the health sector), the vast majority of the included studies were linked to health, which is likely a reflection of both (i) that the KT literature is most advanced in the health sector, and (ii) based on the choice of the databases used.

From the CIS process and stakeholder dialogues, the authors were able to extract information on (i) the definitions and theories most linked to the research debate of EIP institutionalization; (ii) the domains of EIP institutionalization, representing the “building blocks“ and core components of institutionalization; and (iii) the process of EIP institutionalization, representing stages of maturation in the EIP journey. Some key guiding principles/values that support institutionalization were also identified and are further explored in Additional file 4.

### Definition and theory of institutionalization

The literature on institutionalization is broad, with the concept of institutionalization being described as a big tent [44], encompassing a variety of meanings and underscoring the general lack of consensus and conceptual separation [45]:

- Frozen, stabilized, accepted, sustained, durable, persistent, and maintained (Ledford, referenced in [45]), continued [45, 46], and long-lasting [46], permanent [12],
- Routinized [45, 47–49], and
- “Built-in-ness“ (Miles, referenced in [45]), integrated [12, 50], incorporated [45, 47], embedded [12], integral to an organization [45].

In this review, no clear conceptual separation could be drawn between the concept of institutionalization and sustainability. On the one hand, these terms are frequently used synonymously [48, 49]l on the other hand, sustainability is seen, at times, as subsuming institutionalization [48, 49, 51, 52] or, conversely, is considered as not having the same degree of constancy—for example, new organizational activities can be sustained through temporary supports provided to an organization [12]. Additional overlaps were identified with the concepts of routinization [48, 49], scaling up [53], culture, and—in part—capacity-building [11].

Despite its diverse definitions, the idea common to all usage of the term “institutionalization“ is long-term viability, that is, the establishment of a relatively stable situation throughout time and space. Once in place, new institutions are viewed as fairly resilient, often difficult to change [12, 50]. Over time, institutions can become “locked in“ and path-dependent. Maintaining the same direction is advantageous, while the costs of amending the course increase [54, pp. 144–5; 55]. However, rather than focusing solely on the survival and durability per se, two mutually reinforcing concepts of institutionalization stand out:

- *Legitimacy*, which is understood as “a generalized perception or assumption that the actions of an entity are desirable, proper, or appropriate within some socially constructed system of norms, values, beliefs, and definitions” (Suchman, 1995, p. 574, cited in [44, 54, p. 71]), which is associated with different degrees to which a practice obtains social approval, essential for the institution’s survival [12, 44].
- *Taken-for-grantedness*, which refers to the reproduction of social order through standardized and habitualized behaviours for which meaning has become generalized, integrated and embedded into everyday life, independent of specific individuals who perform the action [12, 44].

Scott proposes three dimensions of institutionalization that reinforce legitimacy and taken-for-grantedness: “Institutions are comprised of regulative, normative, and cultural-cognitive elements that, together with associated activities and resources, provide stability and meaning to social life.” [47, p. 56]. The normative and cultural-cognitive dimensions draw, in part, on March and Olsen’s seminal works on institutional rules and logic [54, p. 65]. All three of Scott’s dimensions were applied by Koon et al. to the context of EIP [56].

The regulative pillar of institutionalization refers to the establishment of rules and policies, a common legal environment, and rewards and sanctions [54] that enforce the use of evidence in policy-making [56]. The normative pillar of institutionalization fosters commitments of actors to behave according to an established moral order embedded in society by appealing to appropriateness and the observance of standards and prescriptions [54], for example the types of evidence to be used in policy decision-making [56]. Finally, the cultural-cognitive pillar emphasizes that institutions are social constructions of reality, shaped through interactive and discursive processes providing cognitive frames that determine sense-making and leading to collective beliefs, symbols, identities and taken-for-granted assumptions [54] with regard to the use of evidence in policy-making [54, 56].

Empirically, for institutionalization generally, different constellations of these three pillars can be observed, depending on the circumstances, frequently with one of the pillars taking primacy in view of maintaining social order [54, pp. 170-1f]. For example, equal opportunity in the workplace and the related reform processes in the 1960s were determined by personnel professionals and hence driven by normative mechanisms. As Scott [54] demonstrates, the legislature and the courts, in comparison, played a supportive role, endorsing the programmes already widely accepted among leading firms [54, pp. 161–2].

Similarly, Scott, DiMaggio and Powell’s work on institutional isomorphism outlines how units in society (such as organizations or nation states) adopt new practices when exposed to pressures and uncertainties: coercive isomorphism (based on the power of authority such as governmental mandates), normative isomorphism (where formal education, professional networks or the creation of new supportive organizational structures influence change) and mimetic isomorphism (referring to the imitation of more successful organizations) [12, 54, 57].

While institutionalization can be seen as an outcome (where changes are sustained and become a norm), it is increasingly being described as a process of strategic system transformation [29]. This process operates at multiple levels (through bottom-up development and top-down enforcement) [44, 49], requires continuous adaptation and amelioration in response to the needs of the system [29, 49], and is context-specific as well as historically embedded: “Institutions do not emerge in a vacuum; they always challenge, borrow from, and, to varying degrees, displace prior institutions” [54, p. 114].

Agency, power and interest play a major role in institutional change processes [58]. Contrasting neo-institutionalist approaches, Giddens with his structuration theory highlights agency. Actors can effect change or, to the contrary, maintain and reinforce institutions, which are enacted through routines and reproducing practices, leading to a recursive interaction between structure and agency [59]. Central to agency is the notion of institutional entrepreneurs, namely actors who are endowed with sufficient authority and resources to exert influence on others [60], and discursive processes catalysing the diffusion of new ideas and innovations and increasing their legitimization and social cognition [61]. Organizational leaders might, for instance, trigger change through motivational interventions, demonstrating commitment and action, acting as a problem-solver among peers, establishing comprehensive participatory processes, and nurturing a vision and hope [62].

Reflecting the above, we preliminarily define EIP institutionalization as the “process and outcome of (re-)creating, maintaining and reinforcing norms, regulations, and standard practices that, based on collective meaning and values, actions as well as endowment of resources, allow evidence to become—over time—a legitimate and taken-for-granted part of health policy-making”.

### The six domains of EIP institutionalization

**Fig. 2:**
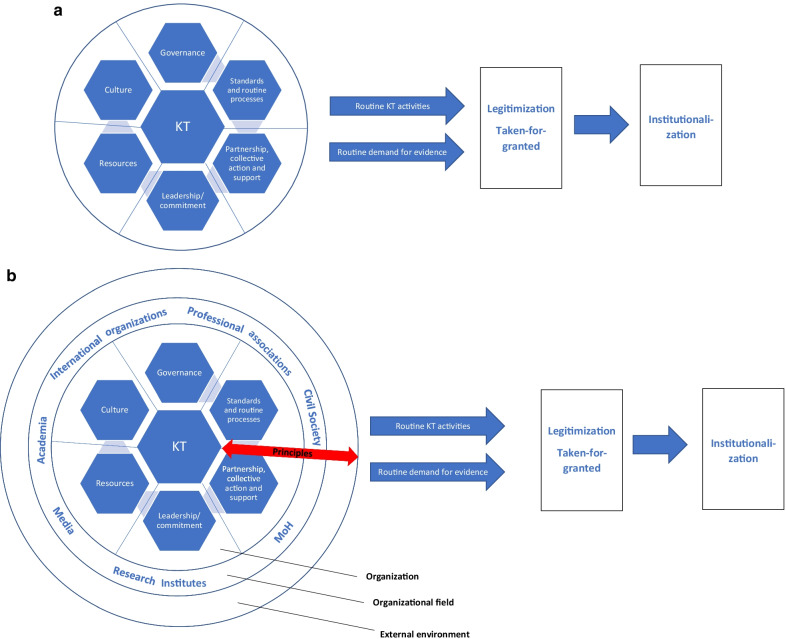
**a** Simple domains framework of EIP institutionalization. **b** Expanded domains framework of EIP institutionalization

Turning to the review question related to the domains of EIP institutionalization, the analysis of the literature and the stakeholder engagement process led to the development of an institutionalization framework (see Fig. 2a, b). We will, in a first step, outline the key domains (the “building blocks“ of EIP institutionalization) and, as a second step, the levels and principles required for the framework to be implemented.

For KT to become a sustained and integral part of health policy-making, routine KT processes and demand for evidence need to be in place [30]. The routine processes enhance the legitimacy and taken-for-grantedness of KT [50], and rely on six key domains (see Fig. 2a).

A summary of the domains is listed below, while the full domains framework and detailed findings can be accessed in Additional file 4.

- Governance: refers to a wide range of rule-making and steering-related functions to achieve EIP institutionalization, including institutionalized structures or platforms that promote interaction and span the boundaries between research and policy [63, 64]. Such platforms not only increase the visibility of KT throughout the system, but also protect it from ad hoc changes in politics and contexts [65].
- Standards and routinized processes: To ensure high-quality KT products and processes that policy-makers trust and hence are more likely to use, standardized processes are required, including tools and protocols [30, 63]. Complemented by well-documented processes, this facilitates the sustainment and institutionalization of KT processes by serving as institutional memory and reducing reliance on individual people with knowledge and skills [63, 66].
- Partnership, collective action and support: Institutionalization is fostered through the extent to which stakeholders interact in the “organizational field“ [59]. Partnerships are essential, as they can foster EIP institutionalization by providing a mechanism for continued engagement and involvement of multiple stakeholders for the same cause, joint problem-solving, identification of resources for ongoing KT and continued technical support [67].
- Leadership and commitment: Strong charismatic leadership is key to creating the conditions for sustained use of evidence [67]. Leaders have the ability to affect the long-lasting adoption of EIP directly, through allocation of resources (human and material), and indirectly, through encouragement, support and mentorship [67, 68].
- Resources: Human, financial, material and information resources are essential inputs for the production and reproduction of social structures over time [30, 69]. In particular, having a critical mass of people, within and outside of the organization, skilful about applying KT routinely and consistently, and throughout time, is a core pillar of EIP institutionalization [67].
- Culture: Culture refers to basic values, assumptions, artefacts and beliefs which are considered valid and are being disseminated and promoted as daily practices [70]. Culture allows for a common understanding of what KT is, what value it can bring about and what is to be expected in terms of activities and benefits [54, 65].

Overall, the domains of governance, standards and routine processes, and culture overlap with Scott’s regulative, normative and cultural-cognitive elements raised in the previous section (see “Definition and theory of institutionalization” section). The first five of the above-listed domains were also raised and elaborated by the stakeholders engaged in this CIS, with a particular emphasis on national institutional structures as well as political will for EIP [42, pp. 21–3, 43, pp. 32–4]. The domain of leadership and commitment reflects the political will aspect; however, the literature points to a more comprehensive domain that also encompasses organizational and distributed leadership and EIP champions needed to initiate and maintain change (see Additional file 4, pp. 13–4). Furthermore, the stakeholder feedback provided more nuanced information on the domain partnership, collective action and support (see Additional file 4, pp. 8–12). During the engagement process, stakeholders emphasized the importance of WHO’s involvement as an external, international health authority providing support and lending legitimacy to the institutionalization process [42, pp. 21–3, 43, pp. 32–4].

As can be seen in Fig. 2b, the framework allows for multiple entry points that need to be considered when designing programmes to support and sustain EIP ([71], see Additional file 4 for details, p. 22). The six domains of institutionalization cut across and need to be implemented at three levels:

- the organizational (e.g. the KTP),
- the organizational field of EIP (or evidence ecosystem), and
- the external environment [72].

The last is the space in which the organizational field of KT/EIP connects with other sectors and institutions which belong to a wider institutional environment. This macro-context encompasses structural political and socioeconomic factors (see Additional file 4 for details, p. 22) which seldom change while influencing how state agencies are using evidence [70]. To institutionalize, one would need to induce work at multiple levels—“top-down“ as well as “bottom-up“ activities—to catalyse longer-term system-wide changes [44] and to allow for the breadth and depth of EIP institutionalization; that is, an innovation needs to be widely adopted and applied to its full potential [47].

The six domains and the three levels of EIP institutionalization are complemented by principled approaches to EIP institutionalization. A range of principles, or values (see Fig. 2b), are proposed, which include inclusive/participatory governance and shared responsibilities [46, 73], evidence-based approach [46, 74], ongoing adaptation, learning and flexibility [34, 62, 74], system thinking [70, 75], credibility [62, 76], legitimacy [44, 62], transparency and accountability [76, 77], independence/autonomy [64, 78] and complexity [79]^4^ (see Additional file 4, pp. 23–4).

EIP institutionalization is the ultimate manifestation of a complex web of interrelations between these six domains and its principles, and is achieved when there is system equilibrium between the components [80]. This equilibrium requires that the domains are aligned and connected [80]. Some domains may be more relevant in certain situations and sociocultural contexts than others. For example, in Brazil, a tendency for coercive mechanisms prevails for initiating social change [81]. Hence, there is no one-size-fits-all approach [65]: institutionalization is historically embedded [59] and context-dependent [65]. The domains framework, therefore, captures both structure and agency—it, on the one hand, provides insights into the existing institutionalized (social) order for EIP while, on the other hand, it allows us to reinforce and maintain change for EIP institutionalization through purposive actions.

### Process of EIP institutionalization and its antecedents

The process of EIP institutionalization is frequently shown as a linear sequence of stages [49, p. 126], each of which is characterized by particular activities and events. Such a model, however, does not take account of the iterative, recursive and reflexive nature of institutionalization and continuous need for adjustments [49, 61].

The process of institutionalization is rather to be seen as circular, with the phases of pre-institutionalization, semi-institutionalization, partial institutionalization and partial de-institutionalization succeeding each other, changing the institutional landscape over time [82]. Stages can, furthermore, interact, overlap or happen simultaneously [83].

**Fig. 3:**
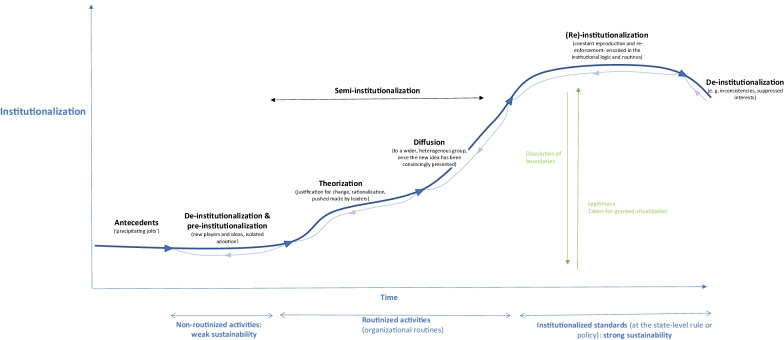
Process framework of EIP institutionalization

When aiming to institutionalize a new practice in an institutional field, existing norms and activities will need to be challenged and de-institutionalized first [12, 84]. The degree of legitimacy and “taken-for-grantedness“ of prevailing institutional practices that are to be replaced need to be examined to ensure that EIP activities can be strategically planned and developed, and that potential resistance can be anticipated [12]. By drawing on existing literature [57, 83–85], we propose an amended five-stage process of institutional change (see Fig. 3):

Precipitating events (antecedents) destabilizing established practices,
De-institutionalization evolving in parallel to pre-institutionalization introducing change processes,
Semi-institutionalization (comprising theorization and diffusion),
(Re)-institutionalization, and
Contradictions, leading to renewed de-institutionalization processes.

Each EIP institutionalization journey, in terms of how evidence has gained profile and becomes embedded and ingrained in societal structures over time, is unique [64].

#### Antecedents

Existing organizational activities and practices that are linked to the organizational core intrinsic values and are taken for granted usually possess a high degree of legitimacy, cultural persistence and endurance [12, 57]. Events that destabilize existing practices and precipitate change, challenge the social consensus of the meaning and value of an institution [57]. Antecedents can lead to sudden changes triggered exogenously, such as legislative changes or a crisis such as COVID-19, or induced through internal sources that may take place over longer time periods [83, 84]. Usually, antecedents that foster the dissipation or rejection of institutional practices and question their legitimacy are classified into three categories [12, 57, 83, 84]:

1. *Political pressures*, such as political interests or the introduction of legal revisions or structural reforms that may challenge the value or political validity and appropriateness of practices which have been reproduced on an ongoing basis;
2. *Functional/technical pressures*, for example, pressure on an organization to adopt innovative mechanisms or performance issues, leading to a decline in utility and instrumental value of the institutionalized practice; and
3. *Social pressures*, for example, through high turnover rates or leadership succession within an organization, weakening the sharing of organizational traditions and interpretative schemes maintaining institutionalized roles and routines [84].

**De-institutionalization** can be defined as “…the delegitimation of an established organizational practice or procedure as a result of organizational challenges to or the failure of organizations to reproduce previously legitimated or taken-for-granted organizational actions.” [84, p. 564]. De-institutionalization may be a conscious and intended step taken by organizations to effect change, or the outcome of processes over which the organization has little influence [84]. Responses to exogenous pressures can vary [12]. Resistance towards antecedents is higher in situations of organizational inertia, while entropy tends to accelerate de-institutionalization [84].

Furthermore, openness and readiness for change within institutions as well as the receptiveness of the environment (what Greenwood and colleagues call “permeability” [57, p. 74]) catalyse de- and pre-institutionalization. This includes pre-existing factors that may have subtly destabilized the institution prior to the emergence of the antecedent [57]. Also, the new practice and its attributes in terms of their degree of sense-making and utility determine the rapidity and depth of the change process.

#### Pre-institutionalization

The steps of de-institutionalization can be seen either as preceding pre-institutionalization [57] or as simultaneous [44]. In the pre-institutionalization phase, new practices, structures and procedures to address the external political, functional and social pressures are in general still idiosyncratic and implemented in an isolated manner [44]. The solutions implemented by other actors may be taken into consideration, possibly through mimetic processes and the establishment of relatively impermanent structures [85] such as temporary institutional KT arrangement piloting the EIP approach to provide proof of concept [86]. In this phase, professional entrepreneurs and experts play an important role in shaping a new discourse and may be called upon to mobilize both material and immaterial assets [60]. In general, the pre-institutionalization phase refers to pragmatic legitimation (i.e. based on self-interest [57]), and testing whether the new practices are seemingly working in a specific given context and begin to make sense [85].

During the **semi-institutionalization** phase (where the practices are increasingly accepted, comprehensible and diffused over time), the particular meanings brought about during the pre-institutional phase are generalized beyond the specific context in which they emerged. This requires, as a first step, theorization, which refers to a formal process of *specification* (making the problem visible) and *justification* of a local solution/innovation to the institutional failure, convincingly narrated and presented to give other actors reason to collaborate by stressing moral legitimacy (by linking it with exiting norms) and/or the pragmatic legitimacy (functional superiority) [57, 85].

As a second step, the innovation is widely diffused among heterogeneous adopters for objectification, that is, for the creation of shared social meaning and collective consensus on the value of the behaviours or arrangements among social actors. The more the arrangement is applied, the more it becomes seen as an appropriate action and obtains cognitive legitimacy. For instance, the provision of an official mandate and formal operationalization of an institutional KT arrangement with clear decision-making processes and procedures might occur in this phase [34]. Such formalization of the KT arrangement will increase the legitimacy of and likely the demand for services to be provided by the institutional KT arrangement, contributing to the repetition, reproduction and re-enactment of specific practices. The structures and rules can, at this stage, still be changed and fairly easily dissolved [85].

While in the pre-institutionalization phase, when diversity and vagueness still dominate, theorization and objectification allow practices to be characterized, codifying them in standards and explicit routines that provide meaning and sense [44], during the (re-)**institutionalization** phase, behaviours and meanings are solidified, encoded in institutional logic and “locked in“ (influenced, e.g., by interest group resistance and support, as well as positive outcomes of the institution), creating resilience and a sustaining momentum/historical continuity through “self-reinforcing feedback dynamics of heightened legitimacy and enhanced taken-for-grantedness” [44, p. 306], as demonstrated by Colyvas and Powell in their seminal work on the institutionalization of technology transfer and the commercialization of university science. Through, for instance, the creation of an Office of Technology at Stanford University, the introduction of specific standardized procedures and routines such as patents and licensing consolidated in the Office of Technology, the clarification of vocabularies and the formalization of role identities, the boundaries between public and private science were redefined and academic entrepreneurship became increasingly legitimate, taken for granted, and institutionalized [44].

Based on Tolbert and Zucker, the complete institutionalization of an innovation “rests on the historical continuity of structure, and especially on its survival across generations of organizational members” [85, p. 184]. According to the domains framework of EIP institutionalization presented in the previous section of this paper, (re-)institutionalization would lead to mature stages of, in an ideal state, all six institutionalization domains described in the previous subsection to provide maximum institutional stability and resilience against new “precipitating jolts“ potentially leading to a process of **de-institutionalization**.

Table 1 integrates the institutionalization domains and process frameworks, and provides a high-level summary of key findings related to the six institutionalization domains across three key phases of institutionalization: pre-, semi- and (re-)institutionalization. The table outlines a set of suggested indicators that reflect low, medium, and high elements of institutional change for EIP, as an input to measure and compare EIP institutionalization across studies. For instance, while in the early stages of institutionalization, external symbols and vocabulary are being borrowed to reflect support, institutional vocabularies manifest and values become clearer during the semi-institutionalized stage, during which resistance of adoption may, however, still prevail. In the re-institutionalization phase, a standardized language and vocabulary with “ready-made categories“ [44, p. 311] will have become socially accepted and taken for granted, an important means to communicate effectively and in a more compressed and precise way. As an example, lengthy elaborations of how to use a fork were at some point obsolete, as everyone knew how to use it [44]. The suggested indicators can be used to highlight and further guide where effort should be focused to catalyse the institutionalization process and promote longevity of KT activities.

**Table 1:** Indicators of EIP institutionalization (building on [44])

Key dimensions/domains of change	Pre-institutionalization	Semi-institutionalization	(Re-)institutionalization
Stage of institutionalization and self-reinforcement	Vulnerable [37]	Anchored [37]	Resilient [37]
Legitimacy and taken-for-grantedness	Low [37]	Medium [37]	High [37]
Governance	• Preliminary institutional KT arrangement [23, 27] with ambiguous role [37]	• Official mandate for an institutional KT arrangement and home [23, 27]^a^ with career opportunities staff [37]^a^ • Varying conventions of the institutional KT arrangement may still trigger debate [37]	• Defined institutional KT arrangement role, steeped with expectations [37], integrated into government planning processes^a^
Standards and routinized processes	• No local standards, familiarization with international tools [71, 72] • Idiosyncratic activities [28, 37]	• Organizational (technical) standards [23]^a^ • Consolidation [37] and organizational routines [23, 42]^a^	• EIP public policy regulation^a^ • Routinized activities at the state level [42] scripted and internalized [37]
Partnership, collective action and support	• Collaboration with selected national champions and stakeholders [27, 73–75] • Strong support from international actors [37, 73, 76] • Success stories/mentoring from more advanced countries [23, 50, 71, 72, 74, 77, 78]	• Collaboration across the evidence ecosystem with an increasing number of key partners • Guidance from international actors and peer support with other countries^a^	• Complex and ongoing multisectoral collaboration across the evidence ecosystem^a^ • Mentoring to other countries^a^
Leadership and commitment	• Scattered (political) leadership and commitment [23, 73, 79, 80]	• Broadening of political support and commitment^a^	Broad-based ecosystem/societal support^a^
Resources	• Learning by doing [37] and training of individuals • Seed funding for EIP activities [23, 27, 73, 81]	• Institutional training [23, 79, 80]^a^ and strong socialization [37] • Securing long-term diversified funding^a^	• Sufficient skilled human resources^a^, and new professions and professional identities emerged [37]^a^ • State budget for EIP and diversified sources of funding^a^
Culture and values	• Relying on external symbols and vocabularies to reflect support [37], including international commitments enhancing legitimacy [71, 78, 82] • Trepidation over adoption requires high articulation [37]	• Institutional vocabularies manifest and values become clearer but can provoke opposition [37] • Technical discussions move from whether or not to do EIP to how to do EIP [37]	• Widely accepted local language and narratives that have become emulated [37]^a^ • Norms and values cemented [37]^a^

^a^Refers to the literature included in the domains framework in Additional file 4

## Discussion

This CIS resulted, as a first step, in a preliminary definition of EIP institutionalization as the “process and outcome of (re-)creating, maintaining and reinforcing norms, regulations, and standard practices that, based on collective meaning and values, actions as well as endowment of resources, allow evidence to become—over time—a legitimate and taken-for-granted part of health policy-making”. While this definition comprehensively encompasses the findings of the analysis of institutionalization theory and definitions, the CIS with its domains and process frameworks goes beyond and has further enriched our understanding by providing insights into the six domains and related principles of EIP institutionalization, as well as the five phases of the EIP institutionalization process.

The domains framework is characterized by six domains that capture both structure and agency, namely (i) governance, (ii) standards and routinized processes, (iii) partnership, collective action and support, (iv) leadership and commitment, (v) resources and (vi) culture—across the institutionalization phases—to ensure maintenance and stability. With the exception of culture, the domains framework reflects the views of stakeholders as expressed during the stakeholder engagement sessions linked to the CIS. We assume that culture, which is the least tangible and visible of the six domains, may in part be “disguised“ by and confounded with other institutionalization factors. Entrenched beliefs and values—as part of the cultural domain—can, for instance, shape practices related to the leadership and commitment domain and influence political commitment and related supportive actions [87], the latter being more easily discernible. Culture, however, is as we postulate and as reflected in the institutionalization definition and frameworks of this CIS, and shown by theoretical institutionalization scholars such as Scott with his conceptual framework of regulative, normative and cultural-cognitive pillars [54], an integral and key element of institutions. Koon et al. [21] also indicate that the development of the cultural-cognitive dimensions of institutionalization is a slow and profound process, which might not be perceptible at its first stages, with greater awareness being put into the normative dimensions.

The proposed process framework reveals EIP institutionalization being achieved through five overlapping process stages: (1) precipitating events (antecedents) destabilizing established practices; (2) de-institutionalization evolving in parallel to pre-institutionalization introducing change processes; (3) semi-institutionalization (comprising theorization and diffusion); (4) (re)-institutionalization; and (5) contradictions, leading to renewed de-institutionalization processes. The KT institutionalization indicators, developed by integrating the domains and process frameworks, reflect the processual aspects of institutionalization as the domains change through time. These indicators may serve to measure and compare EIP institutionalization across future studies. “Full“ institutionalization, however, takes time and considerable efforts. The maturation of the domains also may not all be achieved to the same degree or at the same speed [30, 44].

### Comparison with the literature

This synthesis makes a significant contribution to the knowledge base on EIP institutionalization. Recent reviews have looked at the establishment and sustainment of KTPs [12, 30, 31, 33, 34], considering the potential of institutional theory to advance KT efforts and research [7], as well as investigating the iterative character of policy support organizations that are influenced by political, research and health systems contextual factors [23, 24]. We have further elaborated on these topics and, to our knowledge, our review is the first to address the process, domains and outcomes of EIP institutionalization. Domains such as partnerships, standards, leadership, resources and governance have been highlighted in previous research [22, 24, 25] as key factors for KTP sustainability, but our review has also reflected on interactions across the domains. Specifically, it has been suggested that addressing the features of sustainability, institutionalization and de-institutionalization was a research gap [7], which our review has characterized. Without ensuring that KTPs are embedded in a stable context relying on a range of mature institutionalization domains, the risk remains that a KTP might quickly disappear again due to political changes, resource scarcity or lack of commitment. Reports of a fluid and rather than linear process of EIP institutionalization, with uneven development of different areas from the KTPs, were also found in the literature [20]. For instance, Al Sabahi et al. [26] had already pointed out how different organizations may go back and forth between the different stages even after reaching the maturation stage, repeating processes when a new service or programme is introduced, or major changes are required.

While few scholars have, based on the findings of our review, contributed to the literature of EIP institutionalization or KTP establishment and maintenance, we were able to draw upon a range of papers focusing on health technology assessment and national immunization technical advisory group strengthening and sustainment. The related literature remained, however, overall descriptive in nature. Furthermore, the literature often addresses the stages of institutionalization as disrupting existing structures and processes [12, 57, 83, 84], but instances in which there is a gap, or even a novel opportunity to “layer“ new institutional structures and processes on top of other complementary institutional characteristics, were not accounted for in this CIS. In the context of EIP, it commonly inaugurates a “new way of doing things”, strongly relying on the de-institutionalization stage, but there may be situations where this is not the case.

The process of institutionalizing EIP can also be understood in terms of the sociotechnical transition theory [88], which has been applied to health systems generally [89] and two elements of KT in particular: the development of responsive systematic reviews and public engagement with research [90]. This interpretation sees harsh criticism of healthcare prompting a more scientific approach to testing treatments [91] and niche developments in evidence products, such as systematic reviews in maternity care and critical appraisal skills training programmes accumulating stepwise to create and consolidate the components of a responsive system to meet the evidence needs of decision-makers. In parallel, health services research being challenged by advocates speaking for HIV patients [92], breast cancer patients [93] and maternity service users [94] prompted a transition from governments investing in public understanding of science to investing in co-designing studies with communities [95]. As with institutionalization theory, changing landscapes and shock events destabilize current ways of working, thereby providing a window of opportunity for niche developments to break through and transform systems to align with newly emerging priorities.

### Strengths and limitations

- Strengths
  - A key strength of this synthesis is that methodologies similar to those employed by systematic reviews were applied with its structured and systematic electronic search. A comprehensive search was undertaken with no language restrictions. We ensured that our process was systematic, transparent, robust and aligned with other examples of CISs by having at least two researchers independently conduct each stage.
  - This systematic, structured approach was combined with a qualitative tradition of enquiry with purposive sampling of the literature to fill conceptual gaps and an iterative approach to the analysis.
  - This approach enabled us to overcome some of the inherent challenges which prevail when addressing a broad and complex research question, for which only a sparse and diverse body of literature is available, and allowed us to include both empirical and non-empirical literature to gain important insights for the framework development. In addition, supplementing the synthesis with stakeholder engagement allowed us to test the framework and triangulate findings, albeit with stakeholders linked to EVIPNet and chosen for their understanding of EIP and ability to champion change.
- Limitations
  - One potential limitation is that, given the nascent interest in EIP institutionalization in recent years, new articles may have been overlooked (although this is also the case for other publications).
  - Our methods were systematic and independently verifiable; however, a different study group aiming to respond to the same research question would have identified a different range of primary sources and made different judgements about the relevance, and developed a different framework [72].
  - The proposed frameworks have not been tested, and prospective and retrospective assessments of EIP institutionalization are needed, both with early adopters of EIP institutionalization and those who have made less progress, and those who have developed domains for EIP institutionalization independently of EVIPNet.

Several policy and practice implications can be derived from our analysis: Firstly, EIP institutionalization is complex, with many actors and many interventions coming into play. A comprehensive, transdisciplinary, systematic and transparent system-wide approach that is coordinated between relevant stakeholders is needed, that strengthens both “top-down“ and “bottom-up“ efforts and takes the political dimension—the power, tensions and at times conflicts—into consideration [70]. Secondly, addressing EIP institutionalization will likely be most successful if it is country-driven and owned. There is no one-size-fits-all approach. The approach needs to be built on and adapted to the specific country needs, grounded on the specific country situation. Participatory approaches are required; the approach needs to be owned by all the stakeholders in the system. Thirdly, EIP institutionalization needs to be seen as long-term investment, with a long-term strategy. Reform processes are highly unpredictable, iterative and dependent on the context. Continuous efforts are needed to ensure that progress towards institutionalization is maintained and further strengthened, so that gains are not lost and that potential de-institutionalization tendencies are avoided.

Overall, it is essential that a standardized definition of EIP institutionalization, as suggested in this CIS, is adopted across the evidence ecosystem. This will allow for a common understanding of the complex and multidimensional process of EIP institutionalization and facilitate the operationalization and coordination of related activities. Furthermore, the six domains and the process frameworks, as well as the related indicators presented in this CIS, provide a greater understanding and initial guidance to countries and other actors in the design and implementation of EIP institutionalization, including the measurement of progress.

In terms of research implications, the results of this review also serve as a theoretical construct for researchers to undertake further research and empirical studies on EIP institutionalization, including case studies to validate the frameworks. Also, to date, conceptual and empirical gaps in our understanding of the factors contributing to EIP institutionalization still prevail, and in particular the “weighing“ of different institutionalization domains in specific contexts would be highly valuable to explore. Finally, we would need to strengthen exchange and learning across different systems and countries when it comes to EIP institutionalization and the use of the frameworks presented in this paper.

## Conclusion

The findings of this study provide an evidence-informed framework, triangulated with the views and perspectives of a range of stakeholders, for initiating, strengthening and/or assessing efforts to institutionalize EIP. It can be used as a starting point by both KT researchers and practitioners, including organizational knowledge brokers at national and international levels interested in further understanding the EIP institutionalization process. Our findings indicate the need to design and apply comprehensive, system-wide approaches and the involvement of a multitude of stakeholders to ensure both the breadth and depth of institutionalization. This study contributes to advancing the theoretical and conceptual discussions on EIP institutionalization, just as it provides insights into research gaps regarding the factors that contribute to EIP institutionalization.

## Supplementary Information

**Additional file 1.** Databases and search strategies.

**Additional file 2.** Exclusion and inclusion criteria.

**Additional file 3.** Conceptual mapping.

**Additional file 4.** Domains framework.

## Data Availability

Datasets used during the current study are available from the corresponding author on reasonable request.

## Abbreviations

BIREME: Biblioteca Regional de Medicina (Regional Library of Medicine)
CIS: Critical interpretative synthesis
EIP: Evidence-informed policy-making
EVIPNet: Evidence-informed Policy Network
KT: Knowledge translation
KTP: Knowledge translation platform

## Footnotes

1 WHO defines KT as “the exchange, synthesis, and effective communication of reliable and relevant research results. The focus is on promoting interaction among the producers and users of research, removing the barriers to research use, and tailoring information to different target audiences so that effective interventions are used more widely” [22, p. 140].

2 “Compass“ questions are review questions that provide the overall direction. They allow reviewers to continuously amend the questions throughout the CIS in an iterative manner and therefore serve “as compasses rather than anchors” [37, p. 3].

3 The following countries attended the two EVIPNet Europe multicountry meetings: Austria, Bulgaria, Estonia, Hungary, Lithuania, North Macedonia, Poland, Republic of Moldova, Romania, Serbia, Slovenia, Turkey [42], and Azerbaijan, Georgia, Kazakhstan, Kyrgyzstan, Russian Federation, Tajikistan, Ukraine, Uzbekistan [43].

4 The more complex and interrelated the ecosystem is, the more resilient it is [70].
