# Supplementary material for: Domains and processes for institutionalizing evidence-informed health policy-making: a critical interpretive synthesis
Source: Health Res Policy Syst. 2022 Mar 4;20:27. doi: 10.1186/s12961-022-00820-7 (PMC8894559; doi:10.1186/s12961-022-00820-7)
Supplement: Supplementary file 1 — Additional file 1. Databases and search strategies. [file 12961_2022_820_MOESM1_ESM.pdf]

Additional File 1: Databases and search strategies applied

I.1 PUBMED (search: 6 January 2021), leading to 964 citations

("KT Platform*" OR "Knowledge Translation Platform*" OR "evidence ecosystem*" OR "knowledge ecosystem*" OR (institutionali*[TI] polic*)) OR (((Institutionali* OR sustain* OR routin*) AND ("knowledge Translation" OR "knowledge Use" OR "evidence Use" OR "research Use" OR "knowledge Transfer" OR "research Utilization" OR "knowledge Utilization" OR "evidence Utilization" OR "research Utilisation" OR "knowledge utilisation" OR "evidence utilisation" OR "Diffusion of Innovation"[MH] OR "knowledge Broker*" OR "knowledge dissemination" OR "evidence dissemination" OR "research dissemination" OR "knowledge exchange" OR "knowledge mobilisation" OR "knowledge mobilization" OR "evidence-informed polic*" OR "evidence informed polic*" OR "evidence-based polic*" OR "evidence based polic*" OR "evidence-informed decision*" OR "evidence-based decision*" OR "evidence informed decision*" OR "evidence based decision*" OR "evidence-informed policy-making" OR "evidence informed policymaking" OR "evidence-based policy-making" OR "evidence based policymaking" OR "evidence-informed decision-making" OR "evidence-based decisionmaking" OR "evidence informed decision-making" OR "evidence based decisionmaking" OR "Knowledge Platform*" OR "research-to-policy" OR "research to policy" OR "evidence to policy" OR "evidence-to-policy" OR "science policy" OR "science-policy" OR "policy unit" OR "policy units" OR "advisory bod*" OR "rapid response") AND ("Health Policy"[MH] OR "Public Policy"[MH] OR "Decision Making, Organizational"[MH] OR "decision making" OR "policy making"[MH] OR "Polic*" OR "Policymak*" OR "Policy-mak*")) NOT (mental OR mental* OR elderly OR children OR aged OR psychiatr* OR medical OR clinic*))

I.2 Social Systems Evidence (search: 11 January 2021) leading to 139 citations

("KT Platform" OR "KT Platforms" OR "Knowledge Translation Platform" OR "Knowledge Translation Platforms" OR "Knowledge Platform" OR "Knowledge Platforms" OR "evidence ecosystem" OR "knowledge ecosystem" OR "evidence ecosystems" OR "knowledge ecosystems") OR ((Institutionali* OR sustain* OR routin*) AND ("knowledge Translation" OR "knowledge Use" OR "evidence Use" OR "research Use" OR "knowledge Transfer" OR "research Utilization" OR "knowledge Utilization" OR "evidence Utilization" OR "research Utilisation" OR "knowledge utilisation" OR "evidence utilisation" OR "Diffusion of Innovation" OR "knowledge Broker" OR "knowledge Brokers" OR "knowledge dissemination" OR "evidence dissemination" OR "research dissemination" OR "knowledge exchange" OR "knowledge mobilisation" OR "knowledge mobilization" OR "evidence-informed policy" OR "evidence-informed policies" OR "evidence informed policy" OR "evidence informed policies" OR "evidence-based policy" OR "evidence-based policies" OR "evidence based policy" OR "evidence based policies" OR "evidence-informed decision" OR "evidence-based decision" OR "evidence informed decision" OR "evidence based decision" OR "evidence-informed decisions" OR "evidence-based decisions" OR "evidence informed decisions" OR "evidence based decisions" OR "evidence-informed policy-making" OR "evidence informed policymaking" OR "evidence-based policy-making" OR "evidence based policymaking" OR "evidence-informed decision-making" OR "evidence-based decisionmaking" OR "evidence informed decision-making" OR "evidence based decisionmaking" OR "research-to-policy" OR "research to policy" OR "evidence to policy" OR "evidence-to-policy" OR "science policy" OR "science-policy" OR "policy unit" OR "policy units" OR "advisory body" OR "advisory bodies" OR "rapid response") AND ("Health Policy" OR "Public Policy" OR "Organizational Decision Making" OR "decision making" OR "policy making" OR "Policy" OR policies OR "Policymaking" OR "Policy-making" OR "policy makers" OR "policy maker") NOT (mental OR mental* OR elderly OR children OR aged OR psychiatr*))

I.3 Health Systems Evidence (search: 11 January 2021) leading to 179 citations

("KT Platform" OR "KT Platforms" OR "Knowledge Translation Platform" OR "Knowledge Translation Platforms" OR "Knowledge Platform" OR "Knowledge Platforms" OR "evidence ecosystem" OR "knowledge ecosystem" OR "evidence ecosystems" OR "knowledge ecosystems") OR ((Institutionali* OR sustain* OR routin*) AND ("knowledge Translation" OR "knowledge Use" OR "evidence Use" OR "research Use" OR "knowledge Transfer" OR "research Utilization" OR "knowledge Utilization" OR "evidence Utilization" OR "research Utilisation" OR "knowledge utilisation" OR "evidence

utilisation" OR "Diffusion of Innovation" OR "knowledge Broker" OR "knowledge Brokers" OR "knowledge dissemination" OR "evidence dissemination" OR "research dissemination" OR "knowledge exchange" OR "knowledge mobilisation" OR "knowledge mobilization" OR "evidence-informed policy" OR "evidence-informed policies" OR "evidence informed policy" OR "evidence informed policies" OR "evidence-based policy" OR "evidence-based policies" OR "evidence based policy" OR "evidence based policies" OR "evidence-informed decision" OR "evidence-based decision" OR "evidence informed decision" OR "evidence based decision" OR "evidence-informed decisions" OR "evidence-based decisions" OR "evidence informed decisions" OR "evidence based decisions" OR "evidence-informed policy-making" OR "evidence informed policymaking" OR "evidence-based policy-making" OR "evidence based policymaking" OR "evidence-informed decision-making" OR "evidence-based decisionmaking" OR "evidence informed decision-making" OR "evidence based decisionmaking" OR "research-to-policy" OR "research to policy" OR "evidence to policy" OR "evidence-to-policy" OR "science policy" OR "science-policy" OR "policy unit" OR "policy units" OR "advisory body" OR "advisory bodies" OR "rapid response") AND ("Health Policy" OR "Public Policy" OR "Organizational Decision Making" OR "decision making" OR "policy making" OR "Policy" OR policies OR "Policymaking" OR "Policy-making" OR "policy makers" OR "policy maker") NOT (mental OR mental* OR elderly OR children OR aged OR psychiatr*))

I.4 Virtual Health Library (search: 31 December 2020) leading to 137 citations

(("Knowledge Translation Platform" OR "Knowledge Translation Platforms" OR "institutionalisation of evidence" OR "institutionalization of evidence" OR "institutionalisation of evidence" OR "institutionalization of evidence" OR "institutionalisation of decision making" OR "institutionalization of decision making" OR (TI:institutionali* (polic* OR polit*)) OR "evidence ecosystem" OR "knowledge ecosystems") OR ((Institutional* OR sustain* OR routin* OR instituciona* OR sustentav* OR sustentabilidade OR sostenible* OR rutin* OR rotin* OR "self-sustainability" OR "auto-sustentavel") AND ("traducao do conhecimento" OR "Translational medical research" OR "Diffusion of Innovation" OR "difusão de inovação" OR "Evidence-Based Practice" OR "pratica baseada em evidencia" OR "pratica baseada em evidencias" OR "pesquisa medica translacional" OR "Investigación en Medicina Traslacional" OR "translacion del conocimiento" OR "traducao do conhecimento" OR "traduccion del conocimiento" OR "knowledge Translation" OR "knowledge Use" OR "evidence Use" OR "research Use" OR "knowledge Transfer" OR "research Utilization" OR "knowledge Utilization" OR "evidence Utilization" OR "research Utilisation" OR "knowledge utilisation" OR "evidence utilisation" OR "Diffusion of Innovation" OR "knowledge Broker" OR "knowledge brokers" OR "knowledge dissemination" OR "evidence dissemination" OR "research dissemination" OR "knowledge exchange" OR "knowledge mobilisation" OR "knowledge mobilization" OR "evidence-informed policy" OR "evidence informed policy" OR "evidence-based policy" OR "evidence based policy" OR "evidence-informed decision" OR "evidence-based decision" OR "evidence informed decision" OR "evidence based decision" OR "evidence-informed policy-making" OR "evidence informed policymaking" OR "evidence-based policy-making" OR "evidence based policymaking" OR "evidence-informed decision-making" OR "evidence-based decisionmaking" OR "evidence informed decision-making" OR "evidence based decisionmaking" OR "Knowledge Platform" OR "policy unit" OR "policy units" OR "advisory-body" OR "advisory body" OR "Política Informada por la Evidencia" OR "Evidence-Informed Policy" OR "Política Informada por Evidências") AND ("Health Policy" OR "politica de saude" OR "saude publica" OR "salud publica" OR "politica de salud" OR "Public Policy" OR "Decision Making, Organizational" OR "decision making" OR "policy making" OR Policy OR Policies OR politica* OR policymak* OR "Policy-makers" OR "policy-making" OR "policy making" OR "Decision Making" OR "tomada de decisão" OR "tomada de decisoes" OR "Toma de decision" OR "toma de decisiones"))) AND NOT (Mental OR psiquiatr* OR psychiatr* OR elderly OR children OR idoso* OR criança* OR nino OR ninos OR anciano* OR "adulto mayor" OR "adultos mayores" OR medica* OR clinic*)

I.5 Web of Science (search: 21 January 2021) leading to 378 citations

ALL=("Knowledge Translation Platform" OR "Knowledge Translation Platforms" OR "institutionalisation of evidence" OR "institutionalization of evidence" OR "institutionalisation of evidence" OR "institutionalization of evidence" OR "institutionalisation of decision making" OR "institutionalization of decision making" OR "evidence ecosystem" OR "evidence ecosystems" OR "knowledge ecosystem" OR "knowledge ecosystems") OR (TI=Institutional* OR TI=sustain*

OR TI=routin*) AND (TI=Translation OR TI=translational OR TI=Transfer OR TI=Utilization OR TI=utilisation OR TI="Translational medical research" OR TI="Diffusion of Innovation" OR TI="Evidence-Based Practice" OR TI=Brokers OR TI=Broker OR TI=Informed OR TI=dissemination) AND (AB=Policy OR AB=Policies OR AB="Decision Making" OR AB="Evidence-Informed Policy") AND (AB=Research OR AB=evidence OR AB=knowledge OR AB=information) NOT (AB=Mental OR AB=elderly OR AB=children OR AB=psychiatry OR AB=psychiatric)

I.6 EMBASE (search: 13 January 2021) leading to 230 citations

(("KT Platform" OR "KT Platforms" OR "Knowledge Translation Platform" OR "Knowledge Translation Platforms" OR "evidence ecosystem" OR "evidence ecosystems" OR "knowledge ecosystem" OR "knowledge ecosystems") OR (((Institutionali* OR sustain* OR routin*) AND ("knowledge Translation" OR "knowledge Use" OR "evidence Use" OR "research Use" OR "knowledge Transfer" OR "research Utilization" OR "knowledge Utilization" OR "evidence Utilization" OR "research Utilisation" OR "knowledge utilisation" OR "evidence utilisation" OR "Diffusion of Innovation" OR "knowledge Broker" OR "Knowledge brokers" OR "knowledge dissemination" OR "evidence dissemination" OR "research dissemination" OR "knowledge exchange" OR "knowledge mobilisation" OR "knowledge mobilization" OR "evidence-informed policy" OR "evidence-informed policies" OR "evidence informed policy" OR "evidence informed policies" OR "evidence-based policy" OR "evidence-based policies" OR "evidence based policy" OR "evidence based policies" OR "evidence-informed decision" OR "evidence-informed decisions" OR "evidence-based decision" OR "evidence-based decisions" OR "evidence informed decision" OR "evidence informed decisions" OR "evidence based decision" OR "evidence based decisions" OR "evidence-informed policy-making" OR "evidence informed policymaking" OR "evidence-based policy-making" OR "evidence based policymaking" OR "evidence-informed decision-making" OR "evidence-based decisionmaking" OR "evidence informed decision-making" OR "evidence based decisionmaking" OR "Knowledge Platform" OR "Knowledge Platforms" OR "research-to-policy" OR "research to policy" OR "evidence to policy" OR "evidence-to-policy" OR "science policy" OR "science-policy" OR "policy unit" OR "policy units" OR "advisory body" OR "advisory bodies" OR "rapid response") AND ("Health Policy" OR "Public Policy" OR "Organizational Decision Making" OR "decision making" OR "policy making" OR Policy OR Policymaking OR Policimaker OR policymakers OR "Policy-making")) NOT (mental OR mental* OR elderly OR children OR aged OR psychiatr* OR medical OR clinic*))) AND [embase]/lim NOT [medline]/lim

I.7 CINAHL (search: 21 January 2021) leading to 722 citations

(("Knowledge Translation Platform" OR "Knowledge Translation Platforms" OR "institutionalisation of evidence" OR "institutionalization of evidence" OR "institutionalisation of evidence" OR "institutionalization of evidence" OR "institutionalisation of decision making" OR "institutionalization of decision making" OR "evidence ecosystem" OR "evidence ecosystems" OR "knowledge ecosystem" OR "knowledge ecosystems") OR ((Institutional* OR sustain* OR routin*) AND (Translation OR translational OR Transfer OR Utilization OR utilisation OR "Translational medical research" OR "Diffusion of Innovation" OR "Evidence-Based Practice" OR Brokers OR Broker OR Informed OR dissemination) AND (Policy OR Policies OR "Decision Making" OR "Evidence-Informed Policy") AND TI=(Research OR evidence OR knowledge OR information))) NOT (Mental OR elderly OR children OR psychiatry OR psychiatric)

I.8 Cochrane Library (search: 21 January 2021) leading to 15 citations

("knowledge translation platform" OR "KT Platform" OR "KT Platforms" OR "knowledge translation platforms" OR "institutionalisation of evidence" OR "institutionalisation of evidence" OR "institutionalization of evidence" OR "institutionalisation of decision making" OR "institutionalization of decision making" OR "evidence ecosystem" OR "evidence ecosystems" OR "knowledge ecosystem" OR "knowledge ecosystems") in Title Abstract Keyword OR ("knowledge Translation" OR "knowledge Use" OR "evidence Use" OR "research Use" OR "knowledge Transfer" OR "research Utilization" OR "knowledge Utilization" OR "evidence Utilization" OR "research Utilisation" OR "knowledge utilisation" OR "evidence utilisation" OR "Diffusion of Innovation" OR "knowledge Broker" OR "Knowledge brokers" OR "knowledge dissemination" OR "evidence dissemination" OR "research dissemination" OR "knowledge exchange" OR "knowledge mobilisation" OR "knowledge mobilization" OR "evidence-informed policy" OR "evidence-informed policies" OR "evidence informed policy" OR "evidence informed policies" OR "evidence-based policy" OR "evidence-based

policies" OR "evidence based policy" OR "evidence based policies" OR "evidence-informed decision" OR "evidence-informed decisions" OR "evidence-based decision" OR "evidence-based decisions" OR "evidence informed decision" OR "evidence informed decisions" OR "evidence based decision" OR "evidence based decisions" OR "evidence-informed policy-making" OR "evidence informed policymaking" OR "evidence-based policy-making" OR "evidence based policymaking" OR "evidence-informed decision-making" OR "evidence-based decisionmaking" OR "evidence informed decision-making" OR "evidence based decisionmaking" OR "Knowledge Platform" OR "Knowledge Platforms" OR "research-to-policy" OR "research to policy" OR "evidence to policy" OR "evidence-to-policy" OR "science policy" OR "science-policy" OR "policy unit" OR "policy units" OR "advisory body" OR "advisory bodies" OR "rapid response") in Title Abstract Keyword AND (institutional* OR sustain* OR routin*) in Title Abstract Keyword AND ("Health Policy" OR "Public Policy" OR "Organizational Decision Making" OR "decision making" OR "policy making" OR Policy OR Policymaking OR Policimaker OR policymakers OR "Policy-making")

I.9 Google (search: 21 January 2021) leading to 119 citations

allintitle: (knowledge OR evidence OR research) AND (Translation OR Use OR Transfer OR Utilization OR utilisation) AND (institutionalization OR institutionalisation OR institutional)

I.10 Google scholar (search: 18 January 2021) leading to 118 citations

allintitle: (knowledge OR evidence OR research) AND (Translation OR Use OR Transfer OR Utilization OR utilisation) AND (institutionalization OR institutionalisation OR institutional)
