# Supplementary material for: Domains and processes for institutionalizing evidence-informed health policy-making: a critical interpretive synthesis
Source: Health Res Policy Syst. 2022 Mar 4;20:27. doi: 10.1186/s12961-022-00820-7 (PMC8894559; doi:10.1186/s12961-022-00820-7)
Supplement: Supplementary file 2 — Additional file 2. Exclusion and inclusion criteria. [file 12961_2022_820_MOESM2_ESM.pdf]

Additional File 2: Inclusion and exclusion criteria

A. Inclusion criteria

We included all empirical and non-empirical articles that focus on:

- B.1 and B.2
- B.1 and B.3
- B.1 and B.2 and B.3
- B.4

<u>1st level relevance:</u>

B.1. stands for articles that focus on institutionalization (and its synonyms such as sustainability, routinization, integration, standardization, legitimization, norms, diffusion of innovation, adoption of innovation/system change, scaling-up, resilience).

B.2. stands for articles that focus knowledge translation processes in view of promoting evidence use in policy/evidence-informed policy-making (including Health Technology Assessment (HTA), National Immunization Technical Advisory Groups (NITAGs), National Health Accounts, health information (e.g. health observatories), health impact assessments).

B.3 stands for articles that focus on frameworks (incl models, concepts and/or theory as well as definitions and measures).

B.4 stands for knowledge translation platforms or similar bodies/infrastructure systematically linking evidence to policy and their establishment, functioning and/or institutional capacity (e.g. HTA, observatories, NITAGs, etc.).

B. Exclusion criteria

Articles were excluded based on the following criteria:

1. the translation of research into clinical practice and clinical issues
2. knowledge translation/evidence-informed decision-making (e.g. knowledge translation and other policy support tools, processes, approaches, frameworks etc.) without any link to institutionalization (evidence brief/policy dialogue)
3. efforts exclusively targeting the individual level (e.g. promotion of behavior change)
4. research production (including guidelines and data/health information)/methods (Health Technology Assessment/impact assessment/evaluation)/governance
5. policies/programmes/services (governance/financial/delivery arrangements, including monitoring and evaluation processes) without any link to institutionalization
6. enterprise knowledge ecosystem
7. definitions of brokering, evidence, institutionalization, ecosystem and sustainability other than determined for the Critical Interpretative Synthesis [1]
   (1-4)

[1] In this Critical Interpretative Synthesis (CIS), the following operational definitions were applied:

- Knowledge brokering "is a two-way process that aims to (1) encourage policy-makers to be more responsive to research findings, and (2) stimulate researchers to conduct policy-relevant research and translate their findings to be meaningful to policy-makers." (1, p. 608).
- Evidence is "defined as information – both formal and informal – that can be used in supporting (or otherwise) a conclusion or indicating whether an assumption or proposition is true or valid." (2, p. 3).
- The preliminary operational definition of "institutionalization" for this CIS would be "Routine and sustained government-led and country-owned cycle of commissioning and/or producing, identifying, appraising, synthesizing, packaging and using the best available evidence, embedded in the national policy-making cycle, to systematically and transparently inform national health policy formulation and implementation". (own definition based on (3, p. 11).
- Sustainability is be defined as the capacity of an organization to maintain (i) itself and/or (ii) the programme activities, (iii) the benefits for intended beneficiaries, (iv) a collaborative structure (e.g. coalition) and/or (v) maintaining attention to the issues addressed by the programme once a funded programme has come to closure (4).

The exclusion criteria were iteratively revised and framed more precisely as screening of retrieved articles commences, when it became clearer the types of literature should not be included in the analysis

References

1. Van Kammen J, de Savigny D, Sewankambo N. Using knowledge brokering to promote evidence-based policy-making: the need for support structures. Bulletin of the World Health Organization. 2006;84:608-12.
2. Onwujekwe O, Uguru N, Russo G, Etiaba E, Mbachu C, Mirzoev T, et al. Role and use of evidence in policymaking: an analysis of case studies from the health sector in Nigeria. Health Res Policy Syst. 2015;13:46.
3. World Bank. Where is the money and what are we doing with it? Creating an evidence base for better health financing and greater accountability. A Strategic Guide for the Institutionalization of National Health Accounts. Washington D.C.: World Bank; 2011.
4. Scheirer MA, Hartling G, Hagerman D. Defining sustainability outcomes of health programs: Illustrations from an on-line survey. Eval Program Plann. 2008;31(4):335-46.
