# Supplementary material for: Domains and processes for institutionalizing evidence-informed health policy-making: a critical interpretive synthesis
Source: Health Res Policy Syst. 2022 Mar 4;20:27. doi: 10.1186/s12961-022-00820-7 (PMC8894559; doi:10.1186/s12961-022-00820-7)
Supplement: Supplementary file 3 — Additional file 3. Conceptual mapping. [file 12961_2022_820_MOESM3_ESM.pdf]

Additional File 3: Conceptual mapping template

Global assessment questions:

Does the paper focus on:

1st level relevance[1]

☐ B.1 and B.2, or
☐ B.1 and B.3, or
☐ B.1 and B.2 and B.3
☐ B.4

2nd level relevance

☐ facilitators/barriers of evidence-informed policy-making and relating to networks/institutions/organizations/partnerships
☐ institutionalization/scaling-up, systems and/or organizational learning/change
☐ indirect evidence: partnerships and networks

If for 2nd level relevance, is the paper of high enough relevance to be included into the Critical Interpretative Synthesis?

☐ yes
☐ no
☐ to be checked again later

Conceptual mapping (all boxes that apply are to be checked)

The paper includes:

☐ Institutionalization frameworks
☐ Domains framework
☐ Process framework
☐ Institutionalization theory/ies
☐ Institutionalization definition(s)
☐ Antecedents of institutionalization
☐ Barriers and facilitators of institutionalization

☐ The paper describes in sufficient detail either of the above-mentioned elements relating to institutionalization.

---

[1] As outlined in Additional File 2:

B.1. stands for articles that focus on institutionalization (and its synonyms such as sustainability, routinization, integration, standardization, legitimization, norms, diffusion of innovation, adoption of innovation/system change, scaling-up, resilience).

B.2. stands for articles that focus knowledge translation processes in view of promoting evidence use in policy/evidence-informed policy-making (including Health Technology Assessment (HTA), National Immunization Technical Advisory Groups (NITAGs), National Health Accounts, health information (e.g. health observatories), health impact assessments).

B.3 stands for articles that focus on frameworks (incl models, concepts and/or theory as well as definitions and measures).

B.4 stands for knowledge translation platforms or similar bodies/infrastructure systematically linking evidence to policy and their establishment, functioning and/or institutional capacity (e.g. HTA, observatories, NITAGs, etc.).
