# Supplementary material for: Domains and processes for institutionalizing evidence-informed health policy-making: a critical interpretive synthesis
Source: Health Res Policy Syst. 2022 Mar 4;20:27. doi: 10.1186/s12961-022-00820-7 (PMC8894559; doi:10.1186/s12961-022-00820-7)
Supplement: Supplementary file 4 — Additional file 4. Domains framework. [file 12961_2022_820_MOESM4_ESM.pdf]

Additional File 4: Domains framework

Governance

The governance system is meant to ensure that "…effective processes are in place for decision-making, administration, management, regular communication, conflict resolution and addressing competition for priorities" (1), hence, creating, promoting and monitoring the application of related codes, norms and rules (2). The governance system provides a framework for regular interaction between key actors of the evidence ecosystem with the aim to institutionally strengthen the research-to-policy nexus. Core to EIDM governance are institutional KT arrangements, e.g. Knowledge Translation Platforms, which can be seen as a 'symbolic' and 'relational carrier' fostering the institutionalization of KT (2). Institutional KT arrangements are a clear manifestation of political commitment (3) and can serve as an immaterial "'artefacts' that have the potential to become boundary objects, to which people in different territories and across various boundaries can attach meaning, resonance and value."[1] (1, p. 111). By moving beyond the individual level of relationships, the knowledge broker organization, functioning as a leading referent in charge of KT stewardship with the mandate to support and promote sustainability of evidence use and demand (4).

Sub-domain	Theme description	Examples
**Institutional governance frameworks** (1, 3-30)	Need for a formal governance framework and a collaborative governance strategy, owned by the country, with clear vision/mission statements, measurable short- and long-term objectives, that are aligned with societal goals. ToRs formalize the work of the institutional KT arrangement and its members (allowing for accountability and a clear communication and understanding of its roles and responsibilities), and delineate these from those of other stakeholders in the evidence ecosystem.<br><br>**Facilitators:**<br>● Criteria for developing successful communities of research excellence: Decide on objectives together (22)<br>**Challenges:**<br>● Without a national KT framework it may be difficult to sustain coordination of existing institutional KT	**Governance framework**<br>● Develop a framework that outlines shared definitions of what constitutes collaboration, knowledge on the partners and their roles, commitment to collaboration among stakeholders, an integrated strategy across multiple levels (e.g., provincial, regional and organizational), multiple initiatives based on a mix of top-down enabling and bottom-up innovation, clear ground rules for decision-making and accountability, and well-structured and supported learning networks with effective monitoring and feedback of progress. (9)<br><br>**Measurable goals:**<br>● Goals have to clearly be conceptualized, realistically costed and measurable in order to monitor and evaluate the relationships between inputs, outputs and outcomes. It helps to develop indicators for monitoring and evaluating goals at the inception stage. Beyond their utility in tracking progress, indicators also enable activities, responsibilities and expected outcomes to be transparent to all partners. (22)<br>● Articulate a common vision for what changes are needed (9)

[1] **Boundary spanning objects:** The subject or topic area around which the collaboration is focused also seems to be important for its success. Good' subjects which are regarded as important and specific, with strong evidence of effective interventions and reflecting clear national research priorities, are linked with the success of the collaboration 1. Rycroft-Malone J, Burton C, Wilkinson J, Harvey G, McCormack B, Baker R, et al. Collective action for knowledge mobilisation: a realist evaluation of the Collaborations for Leadership in Applied Health Research and Care. Health Serv Deliv Res. 2015;3(44).

	arrangements (7)<br>● The lack of a strategic plan for short- and long-term work might compromise sustainability (31)<br>● Establishing elaborate EIP procedures (in the form of committees, task forces, or job descriptions), if EIP is required by regulation, can also be a means of 'window dressing' to avoid compliance with real change (32)	**Strategic responsiveness**<br>● Align with and adapt the governance framework to existing goals (changing over time),<br>● Strengthen country ownership and alignment with government programmes (19, 33)<br>● Systematic adaptation of the evidence-based interventions to increase continued fit/compatibility of the interventions with the organization (19)
**Legal mandate/formal decree**<br>(1, 3-6, 11, 12, 20, 23, 26, 29, 30, 34)	Institutionalization requires for the institutional KT arrangement to have an official legal mandate (i.e. a law, a decree or a regulation) providing it with authority and legitimacy in its activities.	**Official mandate of the institutional KT arrangement**<br>● A clear mandate from the government needed to institutionalize EIP (5) and increase the institutional KT arrangement's access to resources (20)<br>● Formal, rule-based structures (8)
**Institutional home embedded into existing structures and processes**<br>(1, 3, 4, 7, 8, 10, 11, 13, 17, 19, 26, 27, 29, 30, 33, 35-46)	A credible, objective and trusted institutional KT arrangement spearheading the routine consideration of evidence in decision-making is a key element of KT institutionalization. The institutional KT arrangement's establishment is context-specific and may change throughout time. There is no one-size-fits all establishment of the institutional KT arrangement. Depending on the local culture and existing structures, different scenarios may initially provide legitimacy. The different scenarios and its advantages/disadvantages should be assessed prior to establishing an institutional KT arrangement and regularly re-assessed to allow for an optimal institutional form and functioning throughout the institutional KT arrangement's life-time.<br><br>**Facilitators:**<br>● Advantage of perceived autonomy and independence from political interests when institutional KT arrangements are hosted by universities (47)	**Institutional KT arrangements**<br>● For a policy unit to be considered institutionalized, it must have a virtual or physical 'head office' in which it is housed (31). This enhances and stabilizes the attention and focus on EIP (4)<br>● A visible and physical NITAG secretariat is essential! (44)<br>● Explore different options for hosting the evidence centre while it is being established to ensure the most suitable organizational arrangement (11) to meet stakeholders' needs and contextual requirements (31)<br>● Establish a government structure for handling KT activities; large units in the health sector, such as major referral hospitals, should be provided with their own KT units (7)<br><br>**Embedment of the institutional KT arrangement into existing (policy) processes and systems**<br>● Well defined scale-up strategy, including developing strategies for integration into existing services (24)<br>● Embed unit activities into standard organizational operations, and add lagging or missing activities (5)<br>● Reinforce institutional integration to promote sustainability and credibility (44)<br>● Institutional KT arrangements in most cases not embedded in routine health sector performance reviews and policy processes (27)<br>● Whether the unit's policy is consistent with the mission and operating procedures of the Ministry of Health (31)

	● Institutional KT arrangements might benefit from the credibility of the hosting organization, including financial and legal structures. However, it may be challenging creating a new entity within a larger organization since it may not always be clear who has to sign off on decisions (11)<br>● Adoption of innovation is facilitated when organizations have a research infrastructure and additional resources, however, if the organizational structure is too formal and centralized or requires too much from individuals, adoption is less likely to be successful (37)<br>● Balancing independence and legitimacy/credibility of the institutional KT arrangement vis-a-vis government (10)<br>● Organizations with dedicated staff, with complex structures, and recognized as having better communication and cohesion than others are more likely to result in higher levels of implementation (1)<br><br>**Barriers:**<br>● Personalized leadership of the institutional KT arrangement increases the vulnerability of the institution in case of turn-over (10, 11)	**Size of the institutional KT arrangement**<br>● The literature suggests between 1-5 secretariat members (26) to 10-14 core staff (26)<br><br>**Membership of the institutional KT arrangement**<br>● Ensure multi- and interdisciplinarity of the institutional KT arrangement: The institutional KT arrangement should consist of staff with a considerable range of skills, expertise and high professional reputation (to ensure national credibility) (26), as well as with lived experience (of working in the sector and/or receiving the relevant services) (11). The institutional KT arrangement can also operate as a consortium of multiple partner organizations (11). Administrative support is important for non-technical aspects (22).<br><br>**Multisectoral Steering Committee/leadership**<br>● The Steering Committee (having the mandate to keep projects on track and escalate issues (1)) should be inclusive (48), and comprise of government and non-government stakeholders (10) with research methods expertise, policy expertise, sector expertise and representation of end user as well as funders (11).<br><br>**Leadership of the institutional KT arrangement**<br>● An influential, respected leader should serve as chair of the institutional KT arrangement signaling that EIP is of high priority and give the organization credibility (11). The leadership of the institutional KT arrangement is proposed to be, e.g., lying with the highest levels in the civil service bureaucracy of a ministry (e.g. Secretary (10)<br>● As with the institutional home, the leadership and staffing requirements may change throughout time (11)<br><br>**Team of the institutional KT arrangement**<br>● The existence of specific, committed actors, and their role and power within the organization are seen as crucial factors for institutionalization processes (8)<br>● Organizations with dedicated staff, with complex structures, and recognized as having better communication and cohesion than others are more likely to result in higher levels of implementation (1)<br><br>**Working groups**<br>● The existence of sub-working groups are reported to be more likely to impact on interorganizational partnership (1)

**Planning, monitoring and evaluation**<br>(3-9, 14, 16, 18, 19, 21, 23-26, 28, 35-37, 41, 42, 44, 49)	Strategic and operational planning through inclusive processes, is key for good governance and management. The strategic and operational plans of the institutional KT arrangement should be context-specific, living documents which directs its functioning and outlines common goals, established agreements around required inputs (including financial and human resources), activities, outputs and outcomes, as well monitoring and evaluation requirements. By regularly assessing its performance, the institutional KT arrangement keeps track that interventions are being implemented as planned, identifies deviations and challenges early on allowing to re-adjust its work, demonstrates learning and accountability, and showcases results and success to its partners.<br><br>**Facilitators:**<br>● International and national tools/guidelines for M&E planning can support the unit and its processes (4)<br>● Monitoring of results can contribute to improving the recognition of the importance of evidence in health planning (41)<br>● Successful implementation requires 'hands on' teamwork, focused monitoring and controls, interim reviews and ongoing feedback mechanisms (28)<br>● Creative and innovative strategies to use existing resources and to secure additional resources can spur a poorly funded partnership forward (22)<br>● Fiscal and operational flexibility to dedicate sufficient resources to emerging priority areas (18)<br><br>**Barriers:**<br>● Lack of mid- and long-term planning for resource mobilization (31)<br>● No agreed set of indicators to measure performance of the	**Strategic operational planning**<br>● Rigorous, participatory planning leads to more significant activities linking the work of the institutional KT arrangement to outcomes (1), and requires a good understanding of the KPT's intended user group(s) and the context (11)<br>● Both informal and formal planning instruments are required (8)<br>● The operational plan should explicitly elaborate its sustainability planning (14)<br><br>**Financial planning**<br>● The institutional KT arrangement needs to develop a long-term financial plan to secure funding for sustainable and scalable results (14), document its needs (e.g. financial, equipment, internet connection) (5) and solicit long-term financial commitments from donors (18)<br><br>**Monitoring and evaluation**<br>● Capture progress from the outset (11) through reflexive monitoring (21), and rapid feedback loops to allow meta-learning (1)<br>● Develop and apply a formal monitoring and evaluation framework with measurable goals for success (22), with performance indicators to understand the value of collaboration with external partners and societal contributions (23, 36), and with intermediate outcome and ultimate outcome indicators (11).<br>● Intermediate outcome evaluation can identify intermediate success or failure, and allows for celebrating accomplishments (1)

	institutional KT arrangement (27)	
**(Perceived) added value of the institutional KT arrangement**<br>(3, 19, 23, 26, 31, 44-46, 50)	Observable regular activities of the institutional KT arrangement and perceived, as well as actual tangible benefits of the institution's work on health decision-making in terms of evidence use, catalyzes support and pragmatic legitimacy of the institutional KT arrangement, stress the recognition of the importance of evidence in health planning/decision-making, and incentivize engagement with the institutional KT arrangement.	● Government's perceptions of whether and how a policy unit is achieving its purpose are key in supporting successful policy unit institutionalization (31)<br>● The program's effect on the health attitudes, perceptions, and behaviors in the area it serves (16)<br>● Take-up of recommendations (26), focus on and demand for research/evidence use within the organizational structure (4), impact on policy and decision making (40, 51) and improved service quality through the unit's activities (5) and positive cases of evidence-informed policies
**Policies, regulations and legislation promoting EIP**<br>(3, 4, 12, 31, 33, 35, 37, 40, 46)	The introduction of regulatory measures mandating the systematic and transparent use of evidence in policy-level decisions, are seen as an important means of institutionalization through legitimate enforcement and legal processes.<br>**Challenges:**<br>● Continued existence of silo structures (33, 40). which filter ideas and create barriers for ideas to circulate, both between sectors and vertically (52)<br>● Unclear policy processes and procedures (12)<br>● Strict control on research dissemination (40)	● Governance structures promote use of evidence; e.g. through regulatory frameworks and systems to foster accountability (4)<br>● Policies, regulations and legislation (constitution, National Development plan, etc), budgets) and accountability mechanisms (Integrated and Comprehensive Monitoring system which includes M&E on EIP) and EIP institutional architecture (33)<br>● Creating legal frameworks that function as permanent rules (13, 14, 25)

Standards and routinized processes

Formal standards allow for a consistent KT process and promote the routine consideration of evidence for policy decisions (12). To ensure high-quality KT products, that policy-makers trust and hence are more likely to use, standardized processes are required, including tools and protocols (30, 31). In their seminal paper on commercialization of science and bridging the boundaries between public and private science, Colyvas and Powel, for instance, refer to patents as standards formalizing and codifying the rules of engagement and promoting routines (53). In the case of EIP, a range of other tools guiding the systematic packaging of evidence as well as promoting engagement exist, such as the evidence brief for policy and policy dialogue. Complemented by well-documented processes, this will facilitate the sustainment and institutionalization of KT processes since serving as institutional memory and reducing reliance on individual people with knowledge and skills (30).

Sub-domain	Theme description	Examples
**Standardization of methods and processes (SoPs)**<br>(3, 4, 6, 11, 24, 29, 30, 35, 37, 39, 40, 46)	Standardized methods (such as operational guidelines for practitioners, manuals etc.) and processes (such as standard operating procedures) help to codify, replicate and routinize processes, ensuring institutional continuity beyond the individual actor	**Guidelines, manuals and best practices**<br>● Provide technical guidance, with best practice case studies (3)<br>● Explicit process designed for evidence gathering, appraisal, synthesis, communication and dissemination (31, 51)<br>● Developing methodological solutions to conduct the stakeholder consultation (3)<br><br>**Standard operating procedures**<br>● Inclusive, transparent (12), formalized and approved SoPs (26) that define explicit processes (35)<br>● Standard operating procedures and routines, consistent with the logic of appropriateness and the organizational process model (8)
**Consistent, routine production of KT activities**<br>(8, 35)	Institutionalization manifests itself through the routine and persistent replication of practices, in which tacit knowledge is captured through patterns of regular interactions.	● Regular production of evidence to support policy-making at country level<br>● Routine generation of high-quality and relevant evidence-informed products (12)
**Quality assurance/control**<br>(1, 3, 4, 12, 13, 24, 26, 27, 38)	Quality assurance ensures that a product or activity meets quality requirements or expectations. Quality assurance increases KT credibility and relies, e.g. on validation testing, routine data collection and analysis, and continuous improvement processes.	**Quality improvement//assurance/control measures**<br>● Quality improvement methods and tools, such as PDSA (Plan, Do, Study, Act - an improvement approach based on achieving incremental, small changes) and audit tools (1)<br>● Protocol for validating evidence-to-policy activities and reports (31)<br><br>**Regular monitoring and evaluation**

		• M&E systems for quality control and performance monitoring (24)<br>• Monitoring to ensure the intervention is being implemented as planned (18)

Partnership, collective action and support

Institutional KT arrangements do not operate in isolation but are situated in and interact with the wider evidence ecosystem. Partnerships provide a formal mechanism for ongoing engagement, joint problem-solving, learning and sharing, as well as provision of financial and technical resources through which activities can, throughout time, be internalized, sustained and institutionalized (54). Successful partnerships empower stakeholders, collectively define directions, promote transparency, build trust, and cater for the needs and values of its members (54). Through regular interactions, mutual understanding, norms and practice, as well as the shared sense of belonging to a common community, can be formed (53). Creating strong linkages and exchange between researchers, policy-makers, and other stakeholders throughout the research production and policy processes, is considered key for effective knowledge translation, however, these require considerable efforts, time and supportive leaders (1, 4). Once consolidated, a KT partnership can itself – collectively or individually, through its members - function as an institutional entrepreneur promoting KT institutionalization and, hence, further generate interest and broaden support (7).

Sub-domain	Theme description	Examples
**Institutional mechanisms for intersectoral engagement/interaction**<br>(1, 4, 12, 19, 22, 39-41)	Establishing interactions and relationships between key stakeholders takes time and needs to be supported by structured processes and institutional mechanisms.<br><br>**Facilitators:**<br>● Complexity of the evidence ecosystem seen as one of the key factors contributing to resilience by (33)<br>● Lessons learned suggest that long-term commitments are key for effective partnerships (39)<br><br>**Challenges**:<br>● The mediating issues of power and competition with regard to the communities of-practice are overlooked in the EIP literature (1)<br>● Complexity of the decision-making architecture based on a high number of institutions with a mandate in EIP that needed to be involved leading to lengthy policy negotiations (10)	**Formal structures and processes of collaboration**<br>● Collaborative governance structures increase the opportunities for interaction, collaboration and integration of the activities of both university and the government (39)<br>● Collaborations are not de facto synergistic, they require structures and processes (including the potential for learning and meta-learning) as well as alignment on the purpose and nature of the collaboration about the nature of the collaboration (1)<br><br>**Communities of practice**<br>● These organized groups of people work together on a particular issue of common interest to encourage joint learning and share experience and information. Developing collective meaning and agree on joint areas of work take time, just as the building of trust and motivation to engage (1)<br><br>**Network coordination**<br>● Small networks can be self-governed, while more extensive collaborations tend to be more effective with a lead organization serving as Secretariat, or with partners represent from a range of background (1), including funders, who perceive the institutional KT arrangement as legitimate to lead the collaboration (45).

**Interdisciplinary collaboration, co-production and relationship building**<br>(1, 3, 5-9, 11, 13, 15, 19-22, 24, 26, 33, 34, 37, 41, 44, 50, 55)	Legitimized processes to coordinate partners based on their comparative advantage and resources enhance the effectiveness of multistakeholder collaborations and lead to value creation, based on the principle that "the best partnerships accomplish more together than each individual organization can achieve on its own" (18). Collaborations are negotiated to collectively agree on shared definition of what constitutes partnership, the priorities and objectives, roles, commitment to collaboration, etc.<br><br>**Facilitators:**<br>● Collaborating with partners and users comprehensively on all facets of the institutional KT arrangement's work and understanding their needs is important to enhance their capability, motivation and opportunity to use evidence in decision-making (16)<br>● Clarity on mutual benefits are essential for effective partnerships (39)Proximity contribute to successful collaborations, including physical proximity to foster better communication and co-ordination (1)<br><br>**Challenges:**<br>● The need to overcome different types of boundaries when establishing EIP partnerships: Organizational, epistemic, semantic, professional and geographical boundaries (1)	**Joint actions and co-production**<br>● These arrangements allows for the teaming up of partners for joint activities, e.g. research grants, which are key to institutionalization (15)<br>● Broad social networks of individuals are associated with adoption (37)<br>● Requires establishing mechanisms to promote and facilitate the development of meaningful collaborations between researchers and implementers. Arrangements that allow researchers to form teams and collaborations with each other and with implementers, to win research grants, and to have the benefits and support of institutional affiliation as they progress in their careers are crucial enablers to IRs institutionalization. (15)<br>● Shared memory: how consistent membership across collaborations can develop mutual respect and willingness to work together, and can promote learning from mistakes (1)<br><br>**Strengths through joint venturing based on comparative advantages**<br>● Institutional arrangements, coordination and networking within and between internal and external stakeholders are necessary (31) to bring together complementary skills and resources (1, 33)<br>● To create interactions and relationships based on comparative advantages of stakeholders can act as catalyzers (4)<br>● Joint venturing" and creating leverage through like-minded partnerships (18)<br><br>**Sharing power through collaboration and continued dialogue**<br>● Surfacing and articulating the different perspectives of all stakeholders around collaboration, knowledge and implementation, including engaging in pre-formative activity and continued dialogue (1)<br>● Stakeholders become more knowledgeable, experienced and empowered (1)<br>● Benefits are shared fairly and broadly (1, 22)<br>● Shared decision-making among stakeholders (19)<br><br>**Trust and relationship building**<br>● Establishing (building and re-building) trusting, functional relations, openness and respect (4); (22) enhances motivation and strengthen partnerships (39)

		● Establishing trusting and functional relations takes time (4)<br>● Trust can be achieved more easily when building on previous experience of working with different organizations (1)
**Communication, Marketing and Diffusion Strategies**<br>(4, 7, 11, 14, 16, 18-20, 22-24, 26, 28, 31, 40, 44, 45, 56)	Successful partnerships are built on mutual understanding. Good internal communication is key to build relationship, guide collaboration and make staff/partners feel included. Good external communication, marketing and diffusion strategies influence stakeholders' and the public's awareness of and willingness to support EIP and the institutional KT arrangement's activities.	**Institutionalized channels of communication**<br>● Strong infrastructure for internal and external communication with consistent and clear messaging is essential during change processes and transitions (14) allowing for the development of close collaboration and trust between researchers, intermediaries and policy-makers. (56)<br>● Advocacy platforms and knowledge sharing infrastructure that contribute to sustaining the political commitment (15)<br><br>**Engagement and regularly communication**<br>● By sending out newsletters, social media or maintaining a website, for instance to inform partners and maintain commitment at strategic level, next to giving oral presentations about the collaborative at local and national events, publications in local media and professional media (PR-instrument) (23)<br>● Communication between collaborators is a significant factor for collaboration success, supported by internal and external communication channels and multiple communication strategies. Physical proximity of team members and the level of technical communication strategies employed within the collaboration are also important. Furthermore, communication has the potential to facilitate sharing and therefore potentially to enable learning (1)<br><br>**Demonstration of results and successes**<br>● One of the key features of sustainability is demonstrating program results (50) through targeted and strategic dissemination and communication of outcomes and lessons learned to stakeholders, decision-makers, and the public (18). This increases the institutional KT arrangement's visibility and understanding by key stakeholders (24) and strengthens public support (14)<br><br>**Collective voice**<br>● Advocate for KT and its need by, e.g. (1) using a collective voice to inform decision-makers and generate interest (18), and (2) through the identification of and working

		through KT champions from the =upper or middle management of an organization, e.g. a politician who has expertise in the health sector and who recognizes the value of using research evidence (7)<br><br>**Adaptation and communication of KT products**<br>● 'Packing' the KT products and communicating them to managers so they are embedded in the day-to-day work of the institution (41)<br>● Use of local language (3)
**International collaboration and support**<br>(1, 3-5, 7, 10-12, 15, 19, 22, 26, 27, 36, 41, 44, 57)	**Challenges:**<br>Barrier: inadequate local leadership support has long been identified as one of many institutional and capacity constraints (35)	**International support and partnerships**<br>● International collaboration (40), with international organizations or declarations/targets being able to foster stability (4)<br>● Partnerships work when the following factors are present: responsiveness of northern partners to southern partner demands (22)<br>● Collaboration with an international organization has led to the creation of a favorable ecosystem for HTA, leading to (1) training workshops,(2) obtaining support for setting-up a local network, (3) obtaining support for the identification of national champions, (4) receiving seed funding, (5) engage/consult with and obtain buy-in from key stakeholders at country level (through the training and awareness raising/advocacy materials) (12)<br>● Donor incentives (4)<br><br>**Global standards and tools**<br>● Access to international resources and tools: A web portal with access to additional various sources of advice and guidance, (e.g. a responsive e-enquiry service, e-discussion forum, blogs etc.) (13, 44, 57)<br><br>**Peer-support and -learning across countries**<br>● South-South collaboration: Move away from' one-way donor dependency' to foster autonomous country networks and/or triangular collaboration (North-South-South) (40)<br>● Potential for creating a database of observatories and individuals who could share information and expertise in the future as part of a global support network (57)

		• Secretariat training and technical skills depend largely on external support levels (26), improved when access to international experts for support and mentoring was available (57)

Leadership and commitment

Leaders, or institutional entrepreneurs can be defined as "…actors who leverage resources to create new or transform existing institutions." (58, p. 68), actors who have the ability and the power to inspire and influence others (34); (1). The importance of leadership is widely acknowledged in the literature. Leadership is the most frequently identified factor for creating change processes (1, p. 15). While less pronounced, it is also seen as one of the key features of sustainment (19); (50). Indeed, leaders, champions and institutional entrepreneurs do not only initiate change but actively contribute to the implementation and maintenance of institutions by three main approaches: "creating a vision for change, mobilising resources and motivating others to achieve and sustain the vision." (59, p. 5). Commitment is defined as the dedication to an issue (e.g. EIP) which can materialize in the demonstration of political leadership or resource allocations (60).

Sub-domain	Theme description	Examples
**KT and network leaderships**<br>(1, 7, 18, 19, 28, 31, 34, 35, 37, 46, 50)	Top and distributed leadership is needed to guide and empower staff, peers and partners; create mutual understanding and an atmosphere of trust; as well as foster overall buy-in and impetus.<br><br>**Facilitator:**<br>● Depending on the systems contexts for leadership (i.e. simple, complicated, complex and chaotic) leadership qualities alter: in clear cause-and-effects environment, a traditional leadership approach (with command and control, well-definite role responsibilities and delegation of work) is effective while in more complex systems, leaders need to rather facilitate and empower, create self-organizing structures, participatory action and continuous assessment (9)<br><br>**Challenges:**<br>● Leadership turnover (24); (47); (35)<br>● Personalized leadership and networks: NKP based on personal leadership making itself vulnerable to the high-turnover rate in Indian institutions (10)<br>● Relying on a hierarchy of top-down leadership may hinder adoption (37)<br>● When leaders lack strong agenda-setting and governance skills and appreciation for the research process themselves, they may not understand the value of evidence nor the time and resources required for staff to engage in these activities (4)	**Provision of direction**<br>● Leadership during a time of change/transition is key (14)<br>● Visible, senior leadership have a key role in the co-ordination of the institutional KT arrangement and its partners (1)<br><br>**Leadership capacity/activities**<br>● both planning and executing strategy (28)<br>● influencing program acceptance" (16)<br>● empowering and engages others through effective management (21)<br>● empowering, creating self-organizing structures, participatory action and continuous assessment is in particular important in more complex systems (9)<br>● actively embracing the expertise of stakeholders and the ability to manage for results (1)<br>● attract additional resources (4, 16)<br>● demonstrating "thought leadership" through training, information sharing and learning/collaborative learning (18)<br>● creating safe learning spaces for new ideas, and adaptive implementation (42)<br><br>**EIP and leadership**<br>● Leaders who value EIP can:<br>● motivate staff to follow their example<br>● institutionalize processes for EIP by applying political commitments to establish support<br>● mobilize additional resources to strengthen EIP (4)<br><br>**Leadership characteristics**<br>● Best et al. suggest that large-system transformation is enabled through alignment between top leadership and distributed leadership because this can reduce cognitive dissonance and facilitate integration between

		intraorganizational boundaries (1)<br>● In the context of institutionalization, in particular distributed (21) and collective leadership (51) seems to be playing a major role.
**Champions**<br>(3, 4, 14, 19, 20, 24, 25, 28, 41)	Champions are field or practice leaders, people who can enable and promote change among professionals and peers.	**Champions as catalyzers for EIP**<br>● Working through KT champions to promote KT - from the upper or middle management – with health expertise and who understands the value of EIP (7)<br>● Need for influential champions to support and strengthen implementation (21) and mobilize resources (14)
**Political interest, support and commitment**<br>(3, 10, 12, 19, 20, 24, 31, 39-41, 46, 60, 61)	The governmental system reflects the value of EIP and demonstrates leadership to create enabling environment for EIP.	**Political support**<br>● Internal and external political environment which influences program funding, initiatives, and acceptance (16)<br>● High-level interest and support from the government (27)<br>● The program has leadership support from outside of the organization (14)<br><br>**Public support**<br>● The program has strong public support (14)Processes that mandate, facilitate or reward civil servants who commission or use research evidence, which includes the creation of specific job descriptions and positions is linked to the commitment and value that leader place on research (62)

Resources

Actors are utilizing resources - human, financial, material resources and information - as they produce and reproduce social structures over time. Resources are an essential cornerstone to promoting, strengthening and institutionalizing EIP mechanisms and processes. Institutionalization requires the continuous planning for, mobilization, strengthening, maintenance and renewal of long-term resources (31); (5); (18). Otherwise, as Sewell describes: "[s]chemas not empowered or regenerated by resources would eventually be abandoned and forgotten, just as resources without cultural schemas to direct their use would eventually dissipate and decay". (Sewell, 1992, p. 13) (2, p. 58).

Sub-domain	Theme description	Examples
**Organizational capacity and human resources**<br>(1, 3-7, 12, 14-16, 19, 21-25, 29-31, 35-37, 39-41, 44, 46, 48, 51, 57, 60)	Capacity refers to the ability of actors (at the individual and organizational level) to plan and implement functions in an effective and effective manner. This relies on both, adequate knowledge, skills and experience of staff, as well as sufficient personnel implementing the work of the institutional KT arrangement.<br><br>**Challenges:**<br>● The frequent turnover of policy-makers constraints the institutional memory within policy-making organizations (52)<br>● Staff of the institutional KT arrangement facing competing demands (19) with staff of the institutional KT arrangement frequently responsible for a wider range of roles (47), time constraints (46, 48); (20, 26) and considering EIP as an add-on, not as an integral element of one's job description and part of a continued process to strengthen EIP (23)	**Individual KT capacity**<br>● Knowledge/skills to:<br>- understand and promote KT (27); (7)<br>- engage with research (12)<br>- access research (37)<br>- use research (4)<br>- recommend actions at the local level through research communication skills (41)<br>- implement the policy-mandated practices<br>● Strong depth and breadth of disciplinary and transdisciplinary knowledge and skills for research generation, social facilitation, and stakeholder engagement. The disciplinary mix varies across institutions and over time (Roux et al. 2015)<br><br>**Adequate staffing, expertise and capacity of the institutional KT arrangement**<br>● A strong Secretariat requires technical skills, (26), and dedicated personnel (27)<br>● Recruit additional sufficient well-trained staff to support the activities of the institutional KT arrangement (5), based on long-term planning for recruitment, retention, management, and development (6)<br>● Potentially contracting in and contracting out certain expertise (35)/ access to external technical expertise (26)
**Capacity building and maintenance in the ecosystem**<br>(1, 3, 4, 6, 7, 12, 15, 17, 19, 22, 24, 25, 27, 33, 35-37, 39-41, 44, 46, 50, 51, 57, 60)	To strengthen EIP capacity (of the evidence producer, intermediary and user) formal and informal knowledge and skill development approaches are needed. These can include	**Training opportunities**<br>**(i) for staff of the institutional KT arrangement**<br>• Making a long-term commitment to all staff for workforce development (26)<br>• Maintenance of workforce skills through continued training,

	learning-by-doing, peer-learning, in-service training, institutional capacity building etc. Move from one-off workshops to more institutionalized trainings, from more individual to organizational capacity building, and to a broader offer of training to different stakeholders.<br>**Facilitators:**<br>● Long-term planning for recruitment, retention, management, and development planning is needed to absorb the negative effects of rapid turn-overs of staff (6)<br>● Institutionalized strategies and mechanisms for capacity building (4): From one-off workshops to more institutionalized trainings, from more individual to organizational capacity building, broader offer of training (33)<br>**Challenges:**<br>● Capacity building programmes frequently do not sufficiently focus on suitable knowledge and skills for evidence use (in particular related to synthesis and dissemination) weakening the impact of training incentives on increasing evidence use (4)<br>● High turnover of staff prevents participation in trainings and the application of new skills (4)<br>● The frequent movement of civil servants within policy contexts limits institutional memory within policymaking organizations (52)	booster training sessions, supervision, and feedback (19)<br>• Training in using tools that support research engagement (4)<br>• Maintenance of workforce skills through continued training, booster training sessions, supervision, and feedback (19)<br>**(ii) for policy-makers' and other stakeholders, including civil society**<br>• KT capacity of researchers producers and users (7), in particular when these actors hold institutional powers, which enable them to change and improve institutional cultures and organizational mechanisms (51), as well as develop mutual understanding with different stakeholders (27)<br>• Training of local decision-makers, opinion leaders, the media, etc. on how to use information produced by the institutional KT arrangement (31)<br>• Evidence literacy across citizens, practitioners, policy-makers and donors (51)<br>• Limited technical and/or EIP capacity at national level (12); (47) beyond the national government in particular, in particular in the civil society and in the research infrastructure (33)<br>**Mentoring, coaching and institutional 'on-the-job-learning' arrangements**<br>• Apprenticeships and mentorship models (6); (4)<br>• Face-to face coaching or mentoring by experienced observatory staff (57)<br>• Joint or adjunct positions of policy-makers in universities to create and strengthen linkages and exchange between research and policy (42)<br>• On the job: (6)<br>**Capacity building of future generations**<br>• Facilitation of participation in master classes or PhD training to upgrade competences (23)<br>• Academic or training programmes to support capacity building (40), including postgraduate trainings and international programs (6)<br>• Building scientific capacities for a new generation of health researchers (38)
**System of evidence supply**<br>(3-6, 12, 26, 33, 40, 47, 51, 57)	Local and international evidence is produced, credible and timely research evidence available.	• Secretariat has access to national/international data and technical expertise (26)<br>• Data access, including real-time data, synthesised data, data from different agencies, having relevant documents

	**Facilitators:**<br>• Digitization of datasets, reports and processes can greatly expedite the speed of access, analysis, dissemination (as long as supporting infrastructure such as electricity, internet, functional computers are present) (4)<br>• Presence of a national statistics unit (not necessarily health specific) in multiple cases has also aided in data analysis and availability of digestible/usable results (4)<br>• Language and database barriers are surpassed (4)<br>**Challenges:**<br>• Insufficient good-quality local data (26)	and data in one place that is accessible to everyone (4)<br>• A virtual forum of expert contacts to whom questions can be directed (57)
**Infrastructure**<br>(6, 24)	Having available infrastructure to support implementation, including physical infrastructure, delivery systems, as well as technical and technological resources	• Electricity, high-speed internet, equipment, functional office spaces (4, 5)<br>• Existing information technology (57)
**Financial resources: Funding stability**<br>(1, 3, 4, 6, 7, 11-17, 19, 20, 22, 24, 26, 27, 31, 33, 35, 47, 48, 50, 51, 57, 60, 61)	Mid- and long-term planning for and mobilization of multiple financial sources (donors, public and private) as well as both domestic and international resources provide a solid basis for a sustained operationalization of the institutional KT arrangement<br>**Facilitator:**<br>• Implementing organizations to have a continued presence in each country to allow for additional funding to capitalize and build on what exists (18)<br>• Policy-makers and the donor community jointly developing solutions for sustainable funding of the institutional KT arrangements (31)<br>**Challenges**<br>• Dependence on funding streams that phase-out, stop, and	**Sustainable, long-term (national/international) funding**<br>• The ongoing generation of work requires committed and regular funding (15) to implement and develop products, for training, and deal with high levels of turnover of the teams (41)<br>• Government/state budget earmarked for the activities of the institutional KT arrangement (31)<br>• Viable financing solutions to be developed by policy-makers and donors to cover operational costs of the institutional KT arrangement (31)<br>• Government policies requiring that a certain percentage/amount of the budget is earmarked towards research just as international declarations and goals (e.g. the Algiers Declaration, World Health Assembly resolutions on health research, and the Bamako Call to Action on Research for Health) (4)<br>**Diversification of financial resources**<br>• Diversification of financial resources to reduce vulnerability and dependence on one source of funding (18)<br>• Exploring the use of a business model for funding KT

	start-up again (18)<br>• Considerable time and financial investment required to develop and sustain relationships with multiple donors (18)	services by the institutional KT arrangement, which is, e.g., successfully used by NICE (7)<br>• "the need for a more integrated research funding system that can provide 'a ladder' for the progression of good collaborative research teams from small-scale intensive projects to test initial research ideas and methodologies to large scale projects that can provide rigorous evidence" (p. vii)(22).<br><br>**Flexible funding**<br>• Endowments are a beneficial funding model for institutional KT arrangement as allowing for longer-term plans and greater financial flexibility (11)

Culture

Culture is described as the range of basic values, assumptions and beliefs which are reflected in day-to-day practices (62). Culture provides social meaning, patterns of thinking, feeling, and behaving (2). In the context of EIP, culture shapes the collective meaning attributed to research and what research means for decision-making processes. This, for instance, materializes in incentive systems put in place to motivate and encourage knowledge translation and evidence use (62); (38); (51).

Sub-domain	Thematic description	Examples
**Organizational culture and climate**<br>(3, 8, 17-20, 28, 36, 37, 41, 49, 51, 55, 62, 63)	The receptive context/climate of an organization is important to incorporate innovations and increase its absorptive capacity for new knowledge. Policies and practices can establish an institutional culture of evidence use.<br><br>**Facilitators:**<br>● A receptive context is characterized by the presence of strong leadership; a clear strategic vision, both for the organization and for scaling up; good management relations; "champions" in critical positions; a climate that is conducive to experimentation and risk-taking; and effective monitoring systems to capture and use important data (25)	**Organizational culture and supportive context**<br>● A supportive context at the of community, organizational and societal levels within a supportive physical environment enables sustainable practice (50)<br>● Promoting an institutional culture of learning from mistakes, rather than one where mistakes are punished, can help overcome a compliance-based system and encourage departments to make use of evidence to actively solve problems (rather than avoid them) (Phillips et al., 2014) (62)<br>● 'Organizational readiness' is defined as a shared psychological state in which organizational members feel committed to implementing an organizational change, and confident in their collective abilities to do so, which contributes to 'implementation effectiveness' (36)<br><br>**Culture of demand and use of evidence**<br>● Top-down institutional value and use of evidence, and bottom-up demand for and supply of evidence to develop a culture for evidence use (51)<br>● Culture of evidence use is a cycle consisting of the following phases: Increased use of evidence in decision-making → Positive cases of EIP demonstrate benefits of using evidence in policy → Research evidence is valued as a resource that improves policies → Greater demand for evidence → Increased use of evidence in decision-making (64)
**Beliefs, values and identity**<br>(1, 9, 28, 34, 36, 48, 52, 60, 62)	Beliefs and values shape in how far evidence is being appreciated as an input into decision-making. The way in which findings and proposals emerging EIP are taken in and interpreted are heavily influenced by dominant policy discourses. Through regular interaction of individuals and active collaboration group identity is created since	**Prevailing narratives and discourses**<br>• Relevance of discourses in both creating institutional stability, and leading to/legitimizing institutional change (65)<br>• Important role of media in serving evidence to policy makers; this probably tells about easy access to information but also about issues attracting attention from

	as individuals create meaning through sense making.<br><br>**Facilitator:**<br>• Individual characteristics such as skills and experience, innovativeness, tolerance of ambiguity, propensity towards risk taking are associated with increased adoption (37)<br><br>**Challenges:**<br>• Respect for and value of expert (senior) opinions or authorities is higher than evidence-based research (40)<br>• Immediate cognitive change in organizations is often overlooked in measurement yet imperative in assessing systemic changes with long gestation periods (35)	citizens and voters and thus gaining value in the eyes of policy makers (57)<br><br>**Shared values and understanding**<br>• The shared understandings, values and links that individuals and groups share engender trust and collaboration (22)<br>• Clear articulation of shared values as a foundation for partnership (9)<br>Collaborations are not de facto synergistic but their success is predicated on some effort to align values, goals and purpose (1)<br><br>**Sense making**<br>• Importance of sense-making since individuals are embedded in systemic relations (56), and of internal frame i.e. the degree to which the policy community agrees on the definition of, causes of, and solutions to the problem (34)<br><br>**Joint identity**<br>• A new group identify is being constructed when moving from the transactional stage to the integrative stage, where resources are merged (22)<br>• Identity is generated over time through negotiations, activities and roles bringing researchers and practitioners together, and the formation of a known 'brand' of the institutional KT arrangement (1)<br>• For institutional activities to be sustained over time, continuing socialization of new organizational entrants is necessary to bring their beliefs and expectations surrounding specific institutional practices into alignment with existing expectations (Schein 1990) (63)
**Interests and norms**<br>(23, 36, 37, 41, 46, 47, 57)	Interest, perceptions and knowledge of managers, as well as current norms, are key elements of sustainability	**New norms for EIP**<br>• Intrinsic use of evidence as 'the right thing to do' (51)<br>• Public norms set out for evidence production and use (31)<br><br>**International normative authorities**<br>• International organizations or declarations/targets can foster stability (4)<br>• World Health Assembly Resolution in 2005 (WHA58.34), 2008 Bamako "Call to Action on Research for Health" and 2013 World Health Report calling for strengthened EIP (55)

**Incentives and motivation**<br>(1, 4, 5, 17, 22, 23, 28, 35, 37, 42)	A mix of positive and negative incentives to be used to motivate staff or stakeholders to engage. Incentives can be internal, based on desires or wishes, such as the desire to impress leadership, or driven by external rewards, such as performance requirements and other accountability mechanisms. encouraging the use of evidence.<br><br>**Challenges:**<br>● Insufficient motivation among policy-makers to learn and change (35)	**Intrinsic motivation**<br>● Professional satisfaction to improve professional practice, enhance credibility and professional standing), (35, 51), and<br>● Building on (22) and celebrating achievements (28)<br><br>**Extrinsic motivation**<br>● Financial and nonfinancial incentives and resources (career pathways, networking with higher level staff, traveling, new responsibilities, promotion structures and increasing salaries) (4, 20, 35, 51)<br><br>**Change in systemic incentives**<br>● Academic publication incentive system undermine research-policy engagements (33)<br>● Electoral incentives to use research evidence along with political costs also bear significant weight (62)

External environment

The organizational field, in terms of the evidence ecosystem, connects with other sectors as well as societal and trans-societal actors and institutions which belong to a wider institutional environment, providing the context of and shaping the institutional KT arrangement and its relationships with key stakeholders (2). The macro-context, as applied in this review, encompasses structural political and economic factors which influence how state agencies are using evidence. Structural macro-level factors seldomly change in a substantial manner and hence can be largely considered outside of the setting of the institutional KT arrangement (62).

**Structural factors**<br>• Extent of democracy/political, academic, media freedom<br>• Extent of development commitment of ruling elite (especially to the poorest)<br>• Extent civil society groups have an input into the making of policy<br>• Extent of political volatility<br>• Extent of conflict or insecurity	Macro- level structures refer to political, economic and socio-cultural context and institutions that shape institutionalization processes of EIP	**Extent of democracy**<br>● Centralized systems where pluralistic debates are less prevalent, there is less need for evidence to justify policy decisions (Liverani et al. 2013:4;Weyrauch, 2016 #13193}<br>● An active civil society (31)<br>● Civil society mobilization: the extent to which grassroots organizations have mobilized to press international and national political authorities to address the issue at the global level (34)<br><br>**Extent of development commitment of ruling elite:**<br>● External socioeconomic and political factors, such as legislation and policy positions, influencing the institutional KT arrangement (31)<br>● Graft and corruption (35)<br>● Restraints on autonomy, patronage and dependence on donor assistance (35)<br><br>**Extent of a politically stable environment:**<br>● Political turmoil (35)<br>● Institutionalization of an institutional KT arrangement requires a stable environment (5)<br>● Workplace environment factors: These include the stability of external socioeconomic and political factors (31)

Principles of KT institutionalization

**Inclusive/participatory governance and shared responsibilities:** Governance is inclusive when it effectively supports the engagement of all stakeholders providing the opportunity for broad participation through and bottom-up approaches which favours shared decision-making, responsibilities, power and local ownership, including community engagement.; and when institutions, processes, and activities are open to, accessible and responsive to all stakeholders and members of society.<br><br>(1, 3, 12, 18, 19, 21, 22, 24, 28, 33, 38, 40, 44, 48, 50)
**Evidence-based approach:** Evidence-informed strategy that is informed by what works, when and in which contexts.<br><br>(9, 21, 24, 25, 50)
Ongoing adaptation, learning and flexibility**:** Incremental approaches, and ensuring that learning occurs by design not chance through the creation of safe learning spaces for new ideas to form from experimentation, reflection, co-learning (including engagement outside the agency), embracing error, and adaptive implementation.<br><br>(1, 3, 5, 9, 14, 16, 18-20, 23, 25, 33, 35, 38, 42, 50, 51, 55, 66)
**System thinking:** Considering the systemic relations, the broader socio-political context and the local context in which knowledge use and implementing processes such as sense making and coalition building.<br><br>(9, 11, 22-25, 33, 38, 45, 50, 52, 56, 62)
**Credibility:** Being trustworthy or believable as an institutional KT arrangement by applying systematic, rigorous and objective methods, including conflict management through declarations of interest forms, and delivering high-quality products.<br><br>(1, 6, 9, 11, 12, 26, 40, 42, 44)
**Transparency and accountability:** Making timely, accurate and relevant information available and accessible to stakeholders and the public, and ensuring that the institutional KT arrangement is answerable for its activities, results and performance, including formal reporting obligations, e.g. to the MoH (26) and reciprocal accountability (21).<br><br>(1, 4, 11, 12, 21, 22, 26, 33, 36, 39, 41)

**Independence/autonomy:** Political autonomy, freedom to publish and independence from funders.<br><br>(4, 11, 44, 48)
**Legitimacy:** Assumption that the practices of an actor are appropriate, desire and in line with collectively accepted beliefs, norms and values.<br><br>(1, 2, 32, 53, 65-67)
**Complexity:** Complexity is the "perceived difficulty of implementation, reflected by duration, scope, radicalness, disruptiveness, centrality, and intricacy an number of steps required to implement" (Damschroder 2009, p. 6) (20). The more complex and interrelated the ecosystem is, the more resilient it is. Stewart et al have identified five dimensions of complexity (33):<br>(i) complexity of actors committed to and involved with EIP,<br>(ii) complexity of activities from the systematic production of evidence production for policy to use,<br>(iii) complexity of sectors (beyond health),<br>(iv) complexity of evidence types for decision-making,<br>(v) complexity of the decision-space for which evidence is sought (33)

References

1. Rycroft-Malone J, Burton C, Wilkinson J, Harvey G, McCormack B, Baker R, et al. Collective action for knowledge mobilisation: a realist evaluation of the Collaborations for Leadership in Applied Health Research and Care. Health Serv Deliv Res. 2015;3(44).
2. Scott WR. Institutions and Organizations. Ideas, Interests, and Identities. Fourth edition, ed. Los Angeles, London, New Delhi, Singapore, Washington DC: SAGE; 2014.
3. Linzalone N, Ballarini A, Piccinelli C, Viliani F, Bianchi F. Institutionalizing Health Impact Assessment: A consultation with experts on the barriers and facilitators to implementing HIA in Italy. J Environ Manage. 2018;218:95-102.
4. Schleiff MJ, Kuan A, Ghaffar A. Comparative analysis of country-level enablers, barriers and recommendations to strengthen institutional capacity for evidence uptake in decision-making. Health Res Policy Syst. 2020;18(1):78.
5. Zida A, Lavis JN, Sewankambo NK, Kouyate B, Ouedraogo S. Evaluating the Process and Extent of Institutionalization: A Case Study of a Rapid Response Unit for Health Policy in Burkina Faso. International Journal of Health Policy and Management. 2017;7(1):15-26.

6. Leelahavarong P, Doungthipsirikul S, Kumluang S, Poonchai A, Kittiratchakool N, Chinnacom D, et al. Health Technology Assessment in Thailand: Institutionalization and Contribution to Healthcare Decision Making: Review of Literature. International Journal of Technology Assessment in Health Care. 2019;35(6):467-73.
7. Basaza R, Kinegyere A, Mutatina B, Sewankambo N. National Framework for the Sustainability of Health Knowledge Translation Initiatives in Uganda. International journal of technology assessment in health care. 2018;34(1):120-8.
8. Gopfert C, Wamsler C, Lang W. A framework for the joint institutionalization of climate change mitigation and adaptation in city administrations. Mitig Adapt Strateg Glob Chang. 2019;24(1):1-21.
9. Herbert C, Best A. It's a matter of values: partnership for innovative change. Healthc Pap. 2011;11(2):31-7; discussion 64-7.
10. Sriram V, Bennett S, Raman VR, Sheikh K. Developing the National Knowledge Platform in India: a policy and institutional analysis. Health Res Policy Syst. 2018;16(1):13.
11. Bazalgette L. A Practical Guide for Establishing an Evidence Centre. London: Alliance for Useful Evidence, Nesta; 2020.
12. Sharma M, Teerawattananon Y, Luz A, Li R, Rattanavipapong W, Dabak S. Institutionalizing Evidence-Informed Priority Setting for Universal Health Coverage: Lessons From Indonesia. Inquiry. 2020;57.
13. Anderson PJ, Yoder S, Fogels E, Krieger G, McLaughlin J. The State of Alaska's early experience with institutionalization of health impact assessment. Int J Circumpolar Health. 2013;72.
14. Moreland-Russell S, Combs T, Polk L, Dexter S. Assessment of the Sustainability Capacity of a Coordinated Approach to Chronic Disease Prevention. J Public Health Manag Pract. 2018;24(4):E17-E24.
15. Vega J, Shroff ZC, Sheikh K, Agyepong IA, Tilahun B, Tangcharoensathien V, et al. Capacity, committed funding and co-production-institutionalizing implementation research in low- and middle-income countries. Health Policy Plan. 2020;35(Supplement_2):ii7-ii8.
16. Schell SF, Luke DA, Schooley MW, Elliott MB, Herbers SH, Mueller NB, et al. Public health program capacity for sustainability: a new framework. Implementation Science. 2013;8(1):15.
17. Wernet SP, Singleton JL. Institutionalization of gerontological curricular change in schools of social work. Gerontology & geriatrics education. 2010;31(1):19-36.
18. Story WT, LeBan K, Altobelli LC, Gebrian B, Hossain J, Lewis J, et al. Institutionalizing community-focused maternal, newborn, and child health strategies to strengthen health systems: A new framework for the Sustainable Development Goal era. Globalization and Health. 2017;13(1):37.
19. Hailemariam M, Bustos T, Montgomery B, Barajas R, Evans LB, Drahota A. Evidence-based intervention sustainability strategies: a systematic review. Implement Sci. 2019;14(1):57.
20. Al Sabahi S, Wilson MG, Lavis JN, El-Jardali F, Moat K, Velez M. Examining and Contextualizing Approaches to Establish Policy Support Organizations - A Critical Interpretive Synthesis. Int J Health Policy Manag. 2020.
21. Cote-Boileau E, Denis JL, Callery B, Sabean M. The unpredictable journeys of spreading, sustaining and scaling healthcare innovations: a scoping review. Health Res Policy Syst. 2019;17(1):84.
22. de-Graft Aikins A, Arhinful DK, Pitchforth E, Ogedegbe G, Allotey P, Agyemang C. Establishing and sustaining research partnerships in Africa: a case study of the UK-Africa Academic Partnership on Chronic Disease. Global Health. 2012;8:29.

23. Jansen MW, De Leeuw E, Hoeijmakers M, De Vries NK. Working at the nexus between public health policy, practice and research. Dynamics of knowledge sharing in The Netherlands. Health research policy and systems. 2012;10:33.
24. Milat AJ, Bauman A, Redman S. Narrative review of models and success factors for scaling up public health interventions. Implement Sci. 2015;10:113.
25. Subramanian S, Naimoli J, Matsubayashi T, Peters DH. Do we have the right models for scaling up health services to achieve the Millennium Development Goals? BMC Health Serv Res. 2011;11:336.
26. Howard N, Walls H, Bell S, Mounier-Jack S. The role of National Immunisation Technical Advisory Groups (NITAGs) in strengthening national vaccine decision-making: A comparative case study of Armenia, Ghana, Indonesia, Nigeria, Senegal and Uganda. Vaccine. 2018;36(37):5536-43.
27. Asamani JA, Nabyonga-Orem J. Knowledge translation in Africa: are the structures in place? Implement Sci Commun. 2020;1(1):111.
28. Shirey MR. Addressing strategy execution challenges to lead sustainable change. The Journal of nursing administration. 2011;41(1):1-4.
29. World Bank. Promoting the institutionalization of national health accounts: a global strategic action plan. Washington D.C.: World Bank; 2010 2010.
30. World Bank. Where is the money and what are we doing with it? Creating an evidence base for better health financing and greater accountability. A Strategic Guide for the Institutionalization of National Health Accounts. Washington D.C.: World Bank; 2011.
31. Zida A, Lavis JN, Sewankambo NK, Kouyate B, Moat K. The factors affecting the institutionalisation of two policy units in Burkina Faso's health system: a case study. Health Research Policy and Systems. 2017;15(1):62.
32. Novotná G, Dobbins M, Henderson J. Institutionalization of evidence-informed practices in healthcare settings. Implementation Science. 2012;7(1).
33. Stewart R, Dayal H, Langer L, van Rooyen C. The evidence ecosystem in South Africa: growing resilience and institutionalisation of evidence use. Palgrave Communications. 2019;5(1).
34. Dodd M, Ivers R, Zwi AB, Rahman A, Jagnoor J. Investigating the process of evidence-informed health policymaking in Bangladesh: a systematic review. Health Policy Plan. 2019;34(6):469-78.
35. Naimoli JF, Saxena S. Realizing their potential to become learning organizations to foster health system resilience: opportunities and challenges for health ministries in low- and middle-income countries. Health Policy Plan. 2018;33(10):1083-95.
36. Duckers ML, Wagner C, Vos L, Groenewegen PP. Understanding organisational development, sustainability, and diffusion of innovations within hospitals participating in a multilevel quality collaborative. Implement Sci. 2011;6:18.
37. Wisdom JP, Chor KH, Hoagwood KE, Horwitz SM. Innovation adoption: a review of theories and constructs. Adm Policy Ment Health. 2014;41(4):480-502.
38. Boes S, Mantwill S, Kaufmann C, Brach M, Bickenbach J, Rubinelli S, et al. Swiss Learning Health System: A national initiative to establish learning cycles for continuous health system improvement. Learn Health Syst. 2018;2(3):e10059.
39. El-Jardali F, Ataya N, Fadlallah R. Changing roles of universities in the era of SDGs: rising up to the global challenge through institutionalising partnerships with governments and communities. Health Res Policy Syst. 162018.
40. MacQuilkan K, Baker P, Downey L, Ruiz F, Chalkidou K, Prinja S, et al. Strengthening health technology assessment systems in the global south: a comparative analysis of the HTA journeys of China, India and South Africa. Glob Health Action. 2018;11(1):1527556.

41. de Oliveira S, Bento AL, Valdes G, de Oliveira STP, de Souza AS, Barreto JOM. [Institutionalizing evidence-based policies in BrazilInstitucionalizacion de las politicas informadas por evidencia]. Rev Panam Salud Publica. 2020;44:e165.
42. Roux DJ, Kingsford RT, Cook CN, Carruthers J, Dickson K, Hockings M. The case for embedding researchers in conservation agencies. Conserv Biol. 2019;33(6):1266-74.
43. Robert G, Greenhalgh T, MacFarlane F, Peacock R. Adopting and assimilating new non-pharmaceutical technologies into health care: a systematic review. J Health Serv Res Policy. 2010;15(4):243-50.
44. Adjagba A, Senouci K, Biellik R, Batmunkh N, Faye PC, Durupt A, et al. Supporting countries in establishing and strengthening NITAGs: lessons learned from 5 years of the SIVAC initiative. Vaccine. 2015;33(5):588-95.
45. Forsberg E-M. Institutionalising ELSA in the moment of breakdown. Life Sci Soc Policy. 2014;10(1).
46. Sanz E, Esnaola, S., Aldasoro, E., Bacigalupe, A. Zuazagoitia, J. Pasos hacia la institucionalización de la evaluación del impacto en salud en España: la percepción de los profesionales de salud pública. An Sist Sanit Navar. 2012;35(3):403-12.
47. El-Jardali F, Lavis J, Moat K, Pantoja T, Ataya N. Capturing lessons learned from evidence-to-policy initiatives through structured reflection. Health research policy and systems. 2014;12:2.
48. Javanparast S, Freeman T, Baum F, Labonté R, Ziersch A, Mackean T, et al. How institutional forces, ideas and actors shaped population health planning in Australian regional primary health care organisations. BMC Public Health. 2018;18(1):383.
49. Leeman J, Baquero B, Bender M, Choy-Brown M, Ko LK, Nilsen P, et al. Advancing the use of organization theory in implementation science. Prev Med. 2019;129S:105832.
50. Harris N, Sandor M. Defining sustainable practice in community-based health promotion: A Delphi study of practitioner perspectives: N. Harris and M. Sandor. Health Promotion Journal of Australia. 2013;24(1):53-60.
51. Langer L, Stewart R, Erasmus Y, de Wet T. Walking the last mile on the long road to evidence-informed development: building capacity to use research evidence. Journal of Development Effectiveness. 2015:1-9.
52. Smith K. Institutional filters: The translation and re-circulation of ideas about health inequalities within policy. Policy & Politics. 2013.
53. Colyvas J, Powell WW. Roads to Institutionalization. Research in Organizational Behavior. 2006;27:305-53.
54. Aarons GA, Hurlburt M, Horwitz SM. Advancing a Conceptual Model of Evidence-Based Practice Implementation in Public Service Sectors. Administration and Policy in Mental Health and Mental Health Services Research. 2011;38(1):4-23.
55. Mansilla C, Herrera CA, Basagoitia A, Pantoja T. The Evidence-Informed Policy Network (EVIPNet) in Chile: lessons learned from a year of coordinated efforts. Revista Panamericana de Salud Pública. 2018;41:e36.
56. Contandriopoulos D, Lemire M, Denis J-L, Tremblay E. Knowledge exchange processes in organizations and policy arenas: a narrative systematic review of the literature. The Milbank quarterly. 2010;88(4):444-83.
57. Aspinall PJ, Jacobson B, Castillo-Salgado C. Establishing and sustaining health observatories serving urbanized populations around the world: scoping study and survey. The European Journal of Public Health. 2016;26(4):681-6.
58. Battilana J, Leca B, Boxenbaum E. How Actors Change Institutions: Towards a Theory of Institutional Entrepreneurship. The Academy of Management Annals. 2009;3(1):65-107.

59. Cai Y, Mehari Y. The Use of Institutional Theory in Higher Education Research. In: Jeroen Huisman MT, editor. Theory and Method in Higher Education Research (Theory and Method in Higher Education Research, Vol 1). Bingley: Emerald Group Publishing Limited; 2015. p. 1-25.
60. Taddese A, Anderson K. 100+ Government Mechanisms to Advance the Use of Data and Evidence in Policymaking: A Landscape Review. 2017.
61. Berman J, Mitambo C, Matanje-Mwagomba B, Khan S, Kachimanga C, Wroe E, et al. Building a knowledge translation platform in Malawi to support evidence-informed health policy. Health Research Policy and Systems. 2015;13(1).
62. Weyrauch V, Echt L, Suliman S. Knowledge into policy: Going beyond 'Context matters'. Oxford INASP; 2016.
63. Oliver C. The Antecedents of Deinstitutionalization. Organization Studies. 1992;13(4):563-88.
64. Varallyay I. Theory of Change for enhancing the capacity of decision-makers to use research evidence. Geneva: Wellcome Trust, World Health Organization, Alliance for Health Policy and Systems Research; 2018.
65. Stassen KR, Smolders R, Leroy P. Sensitizing events as trigger for discursive renewal and institutional change in Flanders' environmental health approach, 1970s-1990s. Environ Health. 2013;12:46.
66. Nielsen J, Mathiassen L, Newell S. Theorization and translation in information technology institutionalization: evidence from Danish home care. MIS Quarterly. 2014;38:165-86.
67. Greenwood R, Hinings CR, Suddaby R. Theorizing Change: The Role of Professional Associations in the Transformation of Institutionalized Fields. Academy of Management Journal. 2002;45(1):58-80.
